# Macrophage-pathogen interactions in infectious diseases: new therapeutic insights from the zebrafish host model

**DOI:** 10.1242/dmm.015594

**Published:** 2014-07

**Authors:** Vincenzo Torraca, Samrah Masud, Herman P. Spaink, Annemarie H. Meijer

**Affiliations:** Institute of Biology, Leiden University, Einsteinweg 55, 2333 CC, Leiden, The Netherlands.

**Keywords:** Leukocyte biology, Innate immunity, Infectious disease, Host-directed therapy, *Mycobacterium*, *Salmonella*, *Burkholderia*, *Staphylococcus*, *Shigella*, *Candida*

## Abstract

Studying macrophage biology in the context of a whole living organism provides unique possibilities to understand the contribution of this extremely dynamic cell subset in the reaction to infections, and has revealed the relevance of cellular and molecular processes that are fundamental to the cell-mediated innate immune response. In particular, various recently established zebrafish infectious disease models are contributing substantially to our understanding of the mechanisms by which different pathogens interact with macrophages and evade host innate immunity. Transgenic zebrafish lines with fluorescently labeled macrophages and other leukocyte populations enable non-invasive imaging at the optically transparent early life stages. Furthermore, there is a continuously expanding availability of vital reporters for subcellular compartments and for probing activation of immune defense mechanisms. These are powerful tools to visualize the activity of phagocytic cells in real time and shed light on the intriguing paradoxical roles of these cells in both limiting infection and supporting the dissemination of intracellular pathogens. This Review will discuss how several bacterial and fungal infection models in zebrafish embryos have led to new insights into the dynamic molecular and cellular mechanisms at play when pathogens encounter host macrophages. We also describe how these insights are inspiring novel therapeutic strategies for infectious disease treatment.

## Introduction

The immune system has evolved through the constant interplay between microbes and their multicellular hosts. Selective forces acting on both sides have driven the evolution of a wide variety of virulence mechanisms in pathogens and alternative control mechanisms in their hosts. *In vivo* modeling of infectious disease is essential for understanding this complexity and translating it into novel therapeutic interventions. The immune system, innate and adaptive, is well-conserved among vertebrates. The zebrafish (*Danio rerio*) offers an optically and genetically accessible vertebrate model to study host-pathogen interactions (Renshaw and Trede, 2012; van der Vaart et al., 2012; Ramakrishnan, 2013). At the embryonic and early larval stages, zebrafish provide the opportunity of studying the relevance of innate immunity in a context where no adaptive response has yet been developed, given that early lymphocytes make their first appearance in 4-day-old larvae and a full adaptive immunity requires several weeks to be mounted (Lam et al., 2004; Page et al., 2013).

Macrophages and neutrophils are the main phagocytic cell types of the innate immune system. Zebrafish models provide unique tools for studying the function of phagocytic cells, and these studies can effectively complement studies in other infectious disease models. Other recent reviews highlighted the use of zebrafish for understanding neutrophil biology (Henry et al., 2013; Shelef et al., 2013). Here, we will discuss six zebrafish models for important human pathogens (*Mycobacterium*, *Salmonella*, *Burkholderia*, *Staphylococcus*, *Shigella* and *Candida*), emphasizing the novel insights that these models have recently provided into macrophage biology and highlighting how this could lead to the finding of new host-derived therapeutic strategies.

## Zebrafish macrophage biology and tools for investigating macrophage function

### Ontogeny and properties of early macrophages in zebrafish

The first macrophage precursors appear in the zebrafish embryo as early as 20 hours post-fertilization (hpf) from the anterior lateral plate mesoderm (Herbomel et al., 1999). Following migration to the yolk sac, they differentiate and either invade the head mesenchyme, where they will later differentiate into microglial cells (the resident macrophages of the brain), or enter the blood circulation (Herbomel et al., 1999; Herbomel et al., 2001). These cells, named primitive macrophages, retain proliferative capability and have been reported to exist in mammals too (Takahashi et al., 1996; Herbomel et al., 1999). They can remove apoptotic cells, are able to sense and respond to invading microbes, and can eradicate non-pathogenic infections. Primitive macrophages readily phagocytose microbes from the blood circulation or when present in tissues. In contrast, neutrophils (which develop slightly later) are less efficient in phagocytosing microbes in the blood, but are potent scavengers of surface-associated bacteria (Colucci-Guyon et al., 2011).

Primitive macrophages are gradually replaced by different lineages of macrophages deriving from definitive hematopoiesis, the process that will produce specialized pluripotent cells with the ability to differentiate into all types of mature blood cells. The first wave of definitive hematopoiesis starts at 24 hpf in the posterior blood island or caudal hematopoietic tissue (CHT) with the differentiation of erythromyeloid progenitors (Bertrand et al., 2007). By 48 hpf, these pluripotent progenitors are replaced with another subset of hematopoietic stem and progenitor cells (HSPCs), now able to also differentiate into the lymphoid lineage. These cells originate from the AGM (aorta, gonads and mesonephros), derived from the lateral posterior mesoderm. After leaving the AGM, they migrate to and nest in the CHT, and will provide the second wave of definitive hematopoiesis (Murayama et al., 2006; Bertrand et al., 2007). Development of HSPCs and their emergence from aortic endothelium is remarkably conserved between zebrafish and mammals (Bertrand et al., 2010; Kissa and Herbomel, 2010; Boisset et al., 2010).

Following the second wave of hematopoiesis, macrophage precursors are released into the circulation and will extravasate to seed tissues throughout the whole body, where they differentiate into tissue macrophages. Starting from 4 days post-fertilization (dpf), the kidney marrow, which is the main hematopoietic tissue of the adult fish, develops and will progressively replace the embryonic hematopoietic system. Another component of the mononuclear phagocyte system is represented by the dendritic cell (DC) population, which is also present in zebrafish larvae and can be detected from 8–12 dpf (Wittamer et al., 2011; Svahn et al., 2013).

The infection studies discussed below, using zebrafish embryo and larval models, do not distinguish macrophages from circulating monocytes. Furthermore, possible functional differences between macrophages from primitive or definitive hematopoietic origins are generally not addressed. For more detailed and comparative descriptions of the processes of hematopoiesis in zebrafish and mammals we refer to other reviews (Stachura and Traver, 2011; Jagannathan-Bogdan and Zon, 2013).

### Macrophage defense mechanisms and subversion by intracellular pathogens

Macrophages sense the presence of infection through microbial-specific molecules and host-derived inflammatory mediators. Their chemoattraction to the site of infection depends largely on the function of G-protein-coupled receptors (Xu et al., 1996; Cotton and Claing, 2009). Scavenger and complement receptors play a major role in phagocytosis (Elomaa et al., 1995), and Toll-like receptors (TLRs), in cooperation with other pattern-recognition receptors (PRRs), initiate the innate immune response (O’Neill et al., 2013). TLRs, found on the cell surface and membranes of vesicular compartments, recognize pathogen- and damage-associated molecular patterns (PAMPs and DAMPs, respectively). Another main class of PRRs, the NOD-like receptors (NLRs), performs the same function in the cytosol (Bertin et al., 1999; Inohara et al., 1999). Some NLRs participate in the assembly of the inflammasome, a multiprotein complex able to activate the caspase-1 cascade, which triggers processing of pro-inflammatory cytokines, such as IL1B (interleukin 1 beta), and full activation of the innate immune response (Martinon et al., 2002).

When engulfed by macrophages, microorganisms are exposed to a number of defense mechanisms within the resulting phagosome and through its subsequent fusion with lysosomes. These include the production of reactive oxygen and nitrogen species (ROS and RNS, respectively) (Minakami and Sumimotoa, 2006; El-Gayar et al., 2003), exposure to antimicrobials, the activity of proteases, and acidification (Schmidtchen et al., 2002; Park et al., 1996; Vandal et al., 2008). Escape from the phagosome triggers septin caging and antibacterial autophagy as additional defense mechanisms (Mostowy et al., 2010; Deretic et al., 2013).

**Fig. 1.**
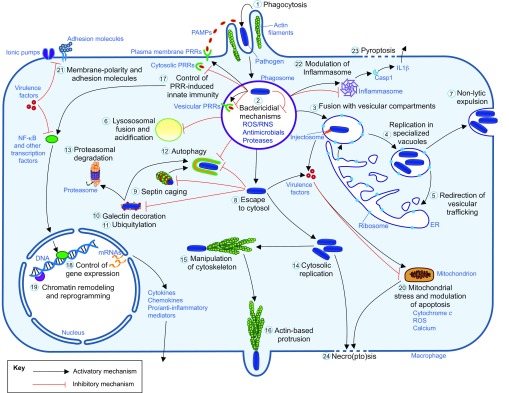
**Evasion of macrophage defense mechanisms by intracellular pathogens.** Upon phagocytosis (1), the pathogens generally reside within phagosomal compartments where a plethora of microbicidal components cooperate in a multidirectional assault to the microbes (2). By transferring virulence factors, often via secretion systems (injectosomes), some pathogens can avoid the classical maturation steps of these compartments, creating a favorable niche for their intracellular growth (3, 4, 5). Fusion of the phagosome with endosomes and/or lysosomes can be blocked (6) and fusion with Golgi- and reticulum-like vesicles can be promoted (3), resulting in the formation of specialized replicative vacuoles (4, 5), in some cases also directed to non-lytic expulsion (7). Several intracellular pathogens are able to escape directly into the cytosol (8). Here, septin cages (9), galectin decoration (10), ubiquitylation (11) and specific routes of antimicrobial autophagy (12) are activated to capture the escapers and redirect them to lytic compartments. Additionally, ubiquitylation of microbial proteins (11) labels these for proteasomal degradation (13). Several intracellular pathogens can efficiently counteract this second line of intracellular defense and replicate freely within the cytosol (14), frequently also manipulating the cell cytoskeleton (15) to sustain their extrusion and dissemination to other host cells (16). Intracellular infections have profound influences also on a wide spectrum of host functions. Cell signaling pathways can be manipulated to modulate the host inflammatory response (17) and control gene expression (18). Some pathogens are also known to induce epigenetic modification of their host cells, leading to reprogramming (19). Some virulence factors directly impact the homeostatic mechanisms by interfering with normal mitochondrial functionality (20), membrane polarity and communication with the extracellular milieu (21). The ultimate possibility for the host to eradicate the infection is to initiate cell (pyroptotic, apoptotic or necroptotic) suicide programs (20, 22, 23, 24). However, the death mechanisms can also be modulated by pathogens, which can benefit from them by the induction of host damage, pathogen dissemination and the initiation of new replicative cycles.

Intracellular pathogens have evolved many strategies to counteract these defenses. These counter-strategies are mediated by virulence factors, which are often secreted directly into the host cell via specialized secretion systems such as the T3SS (type III secretion system) of Gram-negative pathogens and the T7SS (type VII secretion system) of pathogenic mycobacteria (Abdallah et al., 2007; Baxt et al., 2013). Pathogens can also induce significant reprogramming of their host cells through manipulation of signaling pathways and chromatin remodeling; however, these mechanisms are still poorly understood (Masaki et al., 2013; Wang et al., 2005). Intracellular pathogens often block phagosome maturation and fusion with lysosomes or manipulate the vesicular system such that the phagosome is modified to resemble the endoplasmic reticulum or a Golgi-like compartment (Duclos and Desjardins, 2000). Furthermore, several pathogens inject virulence factors that promote actin polymerization to actively stimulate their uptake by both non-phagocytic and phagocytic cells (Ogawa et al., 2008). Pathogens that are able to escape from the phagosome have mechanisms to evade autophagy and can spread from the initially infected cell to other cells by acquiring actin-based motility (Ogawa et al., 2005; Ogawa et al., 2008). Other virulence mechanisms can induce inflammation and different cell-death programs to facilitate the dissemination of infection (Hilbi et al., 1998). These different virulence strategies are schematically depicted in Fig. 1.

### Macrophage markers and transgenic lines

**Fig. 2.**
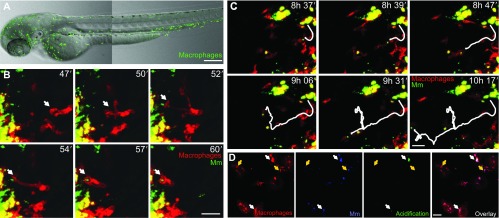
***In vivo* imaging of macrophage responses to infection.** (A) A 3-dpf *Tg(mpeg1:Gal4-VP16/UAS-E1b:Kaede)* zebrafish embryo showing the distribution pattern of macrophages (green). Random patrolling of macrophages is shown in supplementary material Movie 1. (B) Phagocytosis of *M. marinum* (Mm; green) injected into the subcutaneous area overlying a somite in a 2-dpf *Tg(mpeg1:mCherry-F)* embryo. The arrow points at a macrophage (red) in the process of phagocytosis between 47 and 60 minutes post-infection. The images are particulars and stills from supplementary material Movie 2 (10 to 60 minutes post-infection). (C) Macrophage-mediated dissemination of *M. marinum* infection. The white track represents the path of an infected macrophage migrating away from the infection focus. The images are stills and particulars from supplementary material Movie 3, which was taken from the same embryo as in B at a more advanced stage of infection (~8 to ~10 hours post-infection). (D) Partial acidification of phagocytosed *M. marinum*. Bacteria double-labeled with constitutive mCrimson and pH-sensitive green pHrodo are contained within subcellular compartments of macrophages, which are intensely labeled by the membrane-bound mCherry of the *Tg(mpeg1:mCherry-F)* line. White arrows point at bacteria in acidified compartments, where the pHrodo dye is activated. Yellow arrows point at bacteria in non-acidified compartments. Note that most of the intracellular mycobacteria are not acidified, consistent with the ability of this pathogen to counteract phagosome maturation. Macrophages were imaged in the yolk sac circulation valley 5 hours after injection of bacteria into the caudal vein at 2 dpf. Images in A–C were acquired with the Zeiss Observer 6.5.32 laser-scanning confocal, with 10× (A) or 20× (B,C) objectives. Images in D were acquired with Leica TCS SPE confocal with a 20× objective. Figures and movies were processed with ImageJ. The zebrafish transgenic lines *Tg(mpeg1:Gal4-VP16/UAS-E1b:Kaede)* and *Tg(mpeg1:mCherry-F)* were previously described in other reports (Ellett et al., 2011; Bernut et al., 2014). Scale bars: (A) 200 μm; (B,C) 25 μm; (D) 10 μm.

The development of transgenic zebrafish lines with fluorescently labeled leukocytes (supplementary material Table S1) has been key to the successful application of zebrafish for immunological studies. However, until recently, the lack of a specific reporter for the macrophage lineage limited the study of this myeloid subset. This has now been remedied with the development of the *csf1ra* and *mpeg1* reporter lines (Gray et al., 2011; Ellett et al., 2011). These genes are robust markers for macrophages at embryonic and larval stages, because they are co-expressed with the pan-leukocytic marker *lcp1* but not with the neutrophil markers *mpx* and *lyz* (Meijer et al., 2008; Zakrzewska et al., 2010). Despite the fact that *csf1ra* is macrophage-specific within the immune cell types, it is also expressed in neural crest cells and derivatives, such as the xanthophores. Nevertheless, the highly motile macrophages can be distinguished easily from the immobile xanthophores in time-course experiments (Gray et al., 2011). Reporter lines using the *mpeg1* promoter label macrophages but not xanthophores (Fig. 2A; supplementary material Movie 1) and, combined with a neutrophil marker, can show the different kinetics of macrophage and neutrophil responses to infection and wounding, as well as the dynamic interactions between the two cell types (Ellett et al., 2011). The *mpeg1* reporter also labels microglia and it has been suggested to label other antigen-presenting cells, such as the Langerhans dendritic cells, but these could not be detected before 8–9 dpf (Svahn et al., 2013).

Expression of the Gal4 transcription factor under the control of macrophage or neutrophil promoters in combination with a UAS-nitroreductase-mCherry line allows for the specific ablation of one of the two phagocyte populations. This approach can be used to investigate their individual contributions to the immune response and infectious disease pathogenesis (Gray et al., 2011; Prajsnar et al., 2012). Alternatively, *spi1/pu.1* antisense morpholino knockdown can be used to block the development of either macrophages exclusively or of both macrophages and neutrophils, depending on the concentration used (Su et al., 2007). Similarly, *irf8* tools have also been used to deplete specific myeloid cell populations and to skew the development of their progenitors towards macrophages or neutrophils. Morpholino knockdown of *irf8* can completely deplete macrophage differentiation while stimulating an increased output of neutrophils, and *irf8* overexpression can direct myeloid development towards macrophage differentiation (Li et al., 2011).

Many other transgenic lines that label either the entire myeloid population, the early myeloid subset, microglia or all antigen-presenting cells are also very useful for the study of macrophage biology (supplementary material Table S1).

### In vivo visualization of macrophage function

Visualization of live macrophage behavior in zebrafish embryos can be achieved with great structural detail using digitally enhanced differential interference contrast (DIC) microscopy (Herbomel et al., 1999; Davis et al., 2002; Herbomel and Levraud, 2005; Davis and Ramakrishnan, 2009). More recently, there has been tremendous progress in the use of transgenic marker lines (supplementary material Table S1) and labeled pathogens that facilitate live imaging in spatial and temporal dimensions (Fig. 2; supplementary material Movies 2, 3). Photoconvertable fluorescent proteins such as Kaede and Dendra2 have been exploited to show that cells from the CHT can be recruited distally to infection foci and wounds (Yoo et al., 2011), and that mycobacterium-infected macrophages egress from primary granulomas to initiate secondary infection foci (Davis and Ramakrishnan, 2009). For imaging of phagocyte migration, pathogens or specific chemoattractants can be injected subcutaneously or into body cavities such as the otic vesicle and hindbrain ventricle, which can be reached without generating extensive tissue damage, thereby preventing wound-induced leukocyte mobilization (Colucci-Guyon et al., 2011; Benard et al., 2012; Sarris et al., 2012; Yang et al., 2012; Deng et al., 2013; Cambier et al., 2014). To visualize phagocytosis and the intracellular fate of bacteria, the pHrodo dye can be conjugated to bioparticles or to live or heat-killed bacteria (Fig. 2D), providing constitutive fluorescence in one channel and additional fluorescence in another channel following exposure to an acidic environment (Hall et al., 2009). Furthermore, the nature of the compartments where the pathogens reside can be investigated with combinations of different vital stains, most of which are permeable into zebrafish embryos when added to the water. Several pH-sensitive dyes (LysoSensor and LysoTracker) do not distinguish between lysosome-dependent or -independent phagosome acidification mechanisms, but they can be used simultaneously with methods for detection of the activity of lysosomal proteases (MagicRed-Cathepsin and DQ-BSA) (Peri and Nüsslein-Volhard, 2008). Different methods allow *in situ* detection of ROS and RNS responses during infection in zebrafish embryos (Hall et al., 2012; Hall et al., 2013; Bilan et al., 2013; Elks et al., 2013). Also, tools for visualizing ATP, calcium effluxes and apoptosis have been efficiently used in zebrafish (Peri and Nüsslein-Volhard, 2008; Cheung et al., 2011; Sieger et al., 2012). Furthermore, an increasing number of transgenic marker lines for vesicular compartments are becoming available that will help in elucidating the subcellular locations where pathogens reside *in vivo* (supplementary material Table S2).

## New insights into macrophage-pathogen interactions

### Mycobacterium marinum

**Fig. 3.**
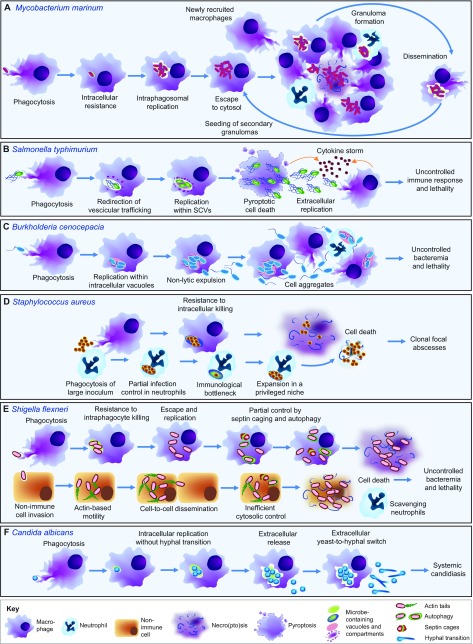
**Models of intracellular infections in zebrafish.** Schematic comparison of the infection phenotypes caused by different pathogens following intravenous injection in zebrafish embryos. (A) *M. marinum* can replicate within phagosomes and also escape into the cytosol. Eventually the host macrophages succumb to the infection, and the pathogen spreads to new macrophages that have been recruited through bacterial virulence mechanisms. This leads to the formation of granulomatous lesions. Occasionally, infected macrophages can egress from the primary granuloma and seed secondary granulomas. (B) *S. typhimurium* avoids the phagosomal defenses by inducing the formation of non-lytic compartments [*Salmonella*-containing vacuoles (SCVs)]. Upon replication, the pathogen induces pyroptotic cell death. Extracellular *Salmonella* continues to replicate. Damage- and pathogen-associated signals contribute to uncontrolled inflammation (‘cytokine storm’), which is rapidly fatal for the host. (C) *B. cenocepacia* can also replicate within intracellular compartments. Additionally, it can be non-lytically expelled from the host macrophages. Within the extracellular environment, the pathogen stimulates leukocyte aggregation and continues replication. The resulting uncontrolled bacteremia is the major cause of the fatal complications. (D) *S. aureus* is largely resistant to intracellular killing when phagocytosed by macrophages and leads to their necrotic death. By contrast, when phagocytosed by neutrophils, most of the pathogen can be efficiently neutralized. However, resistant clones occasionally emerge and expand within these cells. This ‘intraphagocyte niche’ is the reason of the monoclonality of focal staphylococcal abscesses. (E) *S. flexneri* can invade non-immune cells. Within these cells the pathogen escapes immediately into the cytosol and gains actin-based motility, by which it disseminates from cell to cell. Within macrophages, the pathogen can also escape from the phagosome, but here a more efficient cytosolic control partially combats the invader, delaying (although not avoiding) macrophage cell death. Neutrophils represent efficient scavengers for extracellular *Shigella* released from dying epithelial cells and macrophages but the infection is still rapidly lethal. (F) Phagocytosis of *C. albicans* by macrophages leads to a standoff phase, where the host does not degrade the pathogen, but its virulence is contained as it remains locked into a yeast form. The fungus can still slowly replicate and eventually is released. Extracellularly, the yeast can germinate and the resulting fast-replicating hyphae will invade the whole organism, leading to systemic infection.

*M. marinum* is a natural pathogen of zebrafish that causes granulomatous necrotic lesion formation in host tissues. These lesions are histologically very similar to those generated by *Mycobacterium tuberculosis*, the etiological agent of human tuberculosis (Prouty et al., 2003; Swaim et al., 2006). In adult zebrafish, *M. marinum* can cause a latent infection and the bacteria can be reactivated from dormancy by immunosuppressive treatment, as is the case for *M. tuberculosis*, which is estimated to have infected one third of the world population (Parikka et al., 2012; Gengenbacher and Kaufmann, 2012). Tuberculosis therapy is limited by a number of problems, including the poor response of dormant mycobacteria to antibiotics, the increasing prevalence of multidrug-resistant strains and the lack of an effective vaccine against latent or reactivated tuberculosis (Ottenhoff and Kaufmann, 2012). The lack of a mouse model for tuberculosis that fully recapitulates the disease and the risk of working with the human pathogen owing to its airborne transmission emphasize the need for alternative models. The unique accessibility of the early stages of granuloma formation in zebrafish larvae has made the zebrafish-*M. marinum* host-pathogen pair one of the most productive models used to unravel the core pathogenic processes of mycobacterial infections (Davis et al., 2002; Ramakrishnan, 2013; Cronan and Tobin, 2014) (Fig. 3A). Notably, the use of this model has already provided several direct translational applications for human disease treatments (Table 1).

**Table 1.**
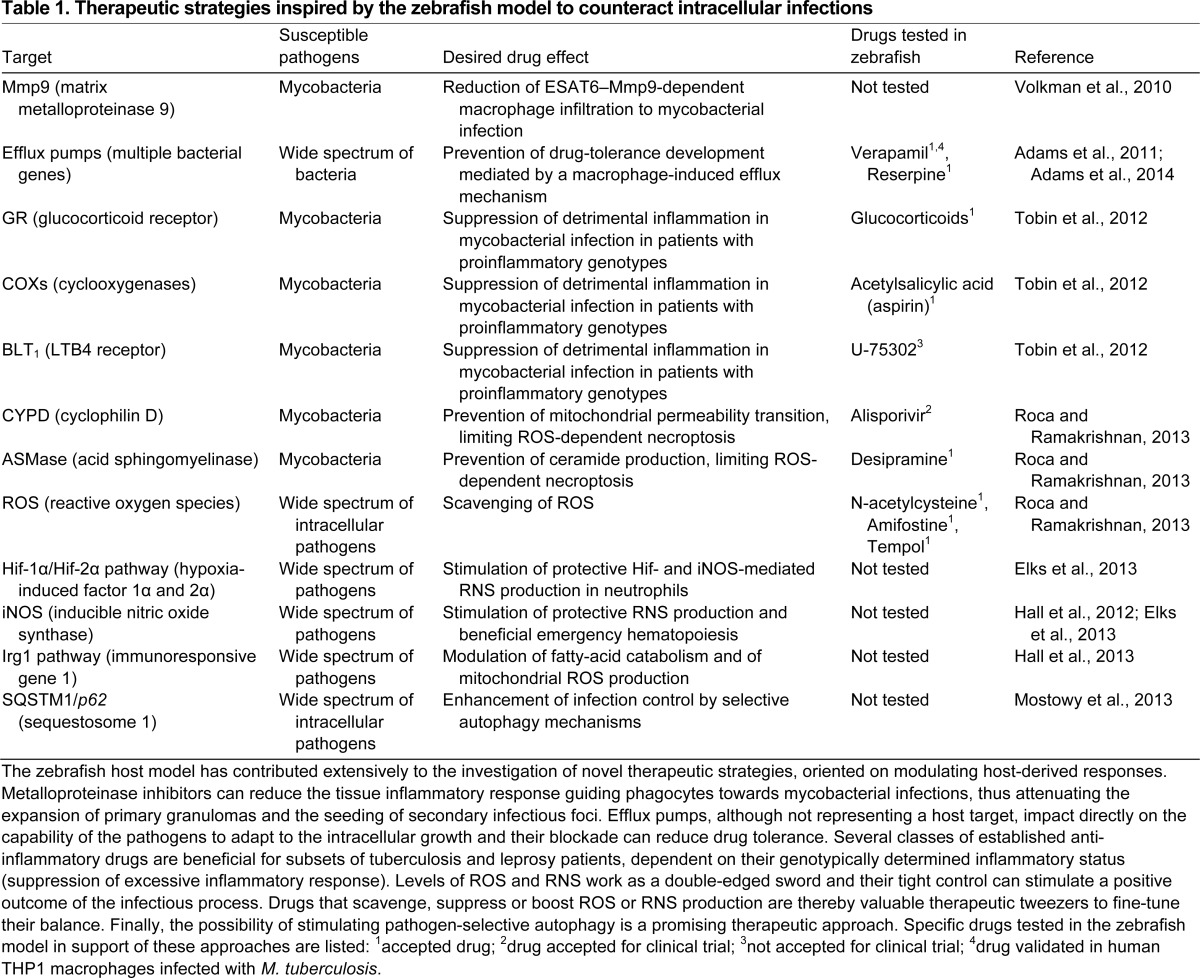
Therapeutic strategies inspired by the zebrafish model to counteract intracellular infections

Infection of zebrafish embryos with *M. marinum* has demonstrated that macrophages are sufficient to initiate granuloma formation in the absence of adaptive immunity (Davis et al., 2002). Subsequently, this model changed the widespread view of granulomas, historically regarded solely as host-protective structures, by showing that early granulomas promote mycobacterial dissemination (Fig. 3A) and that their formation is driven by virulence determinants of the RD1 locus, encoding ESX1, a secretion system conserved in all pathogenic mycobacteria (Volkman et al., 2004; Abdallah et al., 2007; Davis and Ramakrishnan, 2009; Ramakrishnan, 2013). Furthermore, the ESX-1-secreted protein ESAT-6 (Early secreted antigenic target 6) was found to induce matrix metalloproteinase Mmp9 production by epithelial cells surrounding the infection focus, which in turn facilitates macrophage infiltration. As a result, the application of Mmp9 antagonists has been suggested as a host-directed anti-tuberculosis therapy (Volkman et al., 2010) (Table 1). The notion that granulomas are dynamic structures, even during latent infection, is supported by a study of *M. tuberculosis* infection in the macaque model (Lin et al., 2009).

Mycobacteria are well known for developing drug tolerance. The zebrafish embryo model has demonstrated that their intramacrophage localization correlates with development of resistance and that granulomas promote dissemination of this resistant population. Upregulation of bacterial efflux pumps, which are required for intracellular growth, can mediate drug tolerance both in *M. marinum*-infected zebrafish and in *M. tuberculosis*-infected human macrophages. Efflux-pump inhibitors, already available on the market, can reduce this tolerance, and their addition to standard anti-tuberculosis therapy might therefore shorten treatment duration (Adams et al., 2011; Adams et al., 2014) (Table 1).

Another important insight into tuberculosis pathogenesis concerns the relevance of the inflammatory status. A zebrafish mutagenesis screen revealed Lta4h (Leukotriene A_4_ hydrolase) as a host factor that strongly correlates with *M. marinum* susceptibility (Tobin et al., 2010). This enzyme is required for producing LTB_4_ (Leukotriene B_4_), a powerful proinflammatory chemoattractant. Lta4h deficiency correlates with a less inflamed status, due to redirection of its substrates to anti-inflammatory lipoxins, resulting in reduced levels of proinflammatory cytokines such as tumor necrosis factor (TNF). The crucial role of TNF in controlling mycobacterial infection is exemplified by the increased risk of tuberculosis in patients with chronic inflammatory disorders treated with TNF antagonists (Wallis, 2008). However, excessive production of TNF is also associated with higher susceptibility to tuberculosis. This has been shown in zebrafish and other animal models as well as in tuberculosis meningitis patients, where a polymorphism at the *LTA4H* locus that causes increased TNF production has been linked with more progressive disease symptoms (Tsenova et al., 1999; Tobin et al., 2010). Hyper-inflamed and hypo-inflamed statuses have both been associated with necrotic death of infected macrophages and consequent extracellular release of the pathogen (Clay et al., 2008; Roca and Ramakrishnan, 2013). In conditions of low inflammation, macrophages passively undergo necrotic death because they are unable to control intracellular bacterial growth, whereas, in conditions of high inflammation, macrophages actively initiate two ROS-mediated necroptotic pathways, dependent on the activation of the mitochondrial permeability transition pore complex (mPTPC) and of the lysosomal acid sphingomyelinase (aSMase). Drugs targeting these pathways prevent activation of the necroptotic program in the zebrafish model (Roca and Ramakrishnan, 2013). The crucial role of the inflammatory status emphasizes the importance of designing personalized patient therapies: patients with the proinflammatory *LTA4H* genotype might benefit from classical anti-inflammatory drugs [such as corticosteroids or non-steroidal anti-inflammatory drugs (NSAIDs)]; however, these drugs should be avoided in patients with the opposite genotype (Tobin et al., 2010; Tobin et al., 2012). Drugs that directly block the ROS-linked necroptotic pathways will benefit the proinflammatory genotypes without generating detrimental effects on the other genotypes, because the necroptotic pathways are exclusively triggered in hyper-inflamed conditions (Roca and Ramakrishnan, 2013) (Table 1).

The inflammatory response is initiated by TLR recognition of PAMPs. Myd88, a central adaptor in TLR signaling, was recently shown to be required for control of systemic *M. marinum* infection in zebrafish embryos (van der Vaart et al., 2013). In contrast, the initial recruitment of macrophages to a localized *M. marinum* infection in the hindbrain was found to be largely Myd88 independent (Cambier et al., 2014). This effect was linked to the presence of PDIM (phthiocerol dimycoceroserate) lipid on the surface of pathogenic mycobacteria, which masks the underlying PAMPs. Non-pathogenic mycobacteria, which lack PDIM, induce a robust immune response and are efficiently contained. Mutation of the PDIM transporter (*ΔmmpL7*) and of a factor involved in PDIM synthesis (*Δmas*) can restore Myd88-dependent macrophage recruitment and allow an efficient intracellular RNS response against invading bacteria. Interestingly, in the absence of a TLR response, macrophages can still be recruited. This Myd88-independent recruitment was found to be mediated by cell-surface phenolic glycolipids (PGLs), which induce macrophage recruitment through a pathway that is analogous to the mammalian CCL2-CCR2 (CC-motif chemokine ligand–receptor 2) axis. The macrophages recruited in this situation are suggested to be more permissive to intracellular bacterial growth, because they do not drive the strong intracellular RNS response. These observations might also explain why *M. tuberculosis* establishes infection in the lower rather than in the higher respiratory tracts, because the latter is exposed to resident and environmental microbes that can make macrophages more competent for intracellular killing via a continuous transduction of TLR-dependent immune signaling (Cambier et al., 2014).

Although macrophages are the main cell type infected by *M. marinum* following intravenous injection, neutrophils are also important for early infection control in zebrafish. In the early granuloma, the protective role of neutrophils was found to depend on ROS production (Yang et al., 2012). Prior to granuloma formation, neutrophils, both infected and non-infected, also produce RNS (Elks et al., 2013). This RNS production is dependent on inducible nitric oxide synthase (iNOS) and can be modulated by genetic or pharmacological modulation of Hif-α (Hypoxia inducible factor alpha) transcription factors. Increasing Hif-1α signaling or decreasing Hif-2α signaling primes neutrophils with higher levels of RNS prior to infection, thereby limiting susceptibility to mycobacterial infection. Increasing host RNS output by therapeutic targeting of the Hif-α pathway might shift the balance in favor of the host and can thereby be explored as a strategy to complement antibiotic interventions (Table 1).

In addition to the classical microbicidal mechanisms of macrophages, antibacterial autophagy has emerged as an important supplementary control mechanism in mycobacterial infections (Deretic et al., 2013). Using the zebrafish model, colocalization of mycobacteria with the autophagic marker Lc3 has been demonstrated *in vivo* (van der Vaart et al., 2012). Moreover, actintail formation and recruitment of septin cages have been visualized, the latter also being associated with Lc3 and autophagy (Mostowy et al., 2013). The induction of *dram1* (DNA damage-regulated autophagy modulator 1) during infection suggests an immunological function of this autophagy modulator, and the zebrafish model could be further exploited to investigate therapeutic targeting of selective autophagy pathways (Mostowy et al., 2013; Meijer et al., 2014).

Very recently, a zebrafish larval model has also been established to study a rapidly growing mycobacterium, *Mycobacterium abscessus*, an emerging pathogen that causes severe pulmonary infections in individuals with cystic fibrosis (Bernut et al., 2014). The study showed that the virulent rough morphotype of *M. abscessus* is transported to the central nervous system by macrophages, where bacteria released from dying cells form massive amounts of serpentine cords that grow too large to be phagocytosed, leading to acute and lethal infection. Furthermore, *M. tuberculosis* can also be disseminated by zebrafish larval macrophages and is sensitive to antibiotic treatment in this model (Carvalho et al., 2011). It will be of interest to investigate novel therapeutic strategies emerging from the study of *M. marinum* (Table 1) also in the zebrafish models for these human pathogens.

### Salmonella enterica serovar Typhimurium (S. typhimurium)

Like many other Gram-negative enterobacteria, *Salmonella* can infect a diverse range of hosts and cause zoonotic diseases (Fàbrega and Vila, 2013). The *S. enterica* serovar *Typhimurium*, often referred to as *S. typhimurium*, represents a common agent of enteric fever, gastroenteritis and bacteremia, often linked to food poisoning. Although the bacterium is not a natural fish pathogen, zebrafish embryos are strongly susceptible to *S. typhimurium* in experimental settings. Pathogenesis in fish involves some of the acute symptoms seen in humans, including bacteremia and a strong proinflammatory host response (‘cytokine storm’), which are associated with early lethality of the zebrafish embryos (van der Sar et al., 2003; Stockhammer et al., 2009). The life cycle of *S. typhimurium* has been well characterized in infected cell cultures and in mammalian systems: the pathogen is able to alternate phases of intracellular replication within phagocytes and extracellular growth within the damaged tissue (Dunlap et al., 1992; Salcedo et al., 2001). Similar observations have been made in the zebrafish embryo model. *S. typhimurium*, like *M. marinum*, can survive intracellularly in macrophages of zebrafish embryos (Fig. 3B), but infected cells do not disseminate into tissues. Instead, following intravenous injection, the infection remains restricted to the vasculature, with bacteria multiplying both in macrophages and extracellularly at the epithelium of blood vessels (van der Sar et al., 2003). Although infection with wild-type bacteria causes early lethality, bacteria with mutations in lipopolysaccharide (LPS) are attenuated in macrophages and are more sensitive to extracellular lysis, likely due to complement factors (van der Sar et al., 2003).

In sharp contrast with *M. marinum* infection, *S. typhimurium* infection leads to a cytokine storm within hours after intravenous injection (Stockhammer et al., 2009; Ordas et al., 2011; van der Vaart et al., 2012). At the transcriptional level, this response is similar to that observed in infection with the natural fish pathogen *Edwardsiella tarda* (van der Vaart et al., 2013). This proinflammatory transcriptional response provides a useful readout for characterizing the consequences of mutation or knockdown of host genes involved in the immune response. Deficiencies in the TLR signaling components Myd88 and Traf6 strongly reduce expression of transcriptional regulators, signaling components and effectors of the immune response (Stockhammer et al., 2010; van der Vaart et al., 2013). Conversely, these gene groups are hyper-induced following knockdown of the protein tyrosine phosphatase Shp1 (also known as Ptpn6) (Kanwal et al., 2013). These observations are consistent with the function of these genes in mammalian animal models and human patients, where *MYD88* and *TRAF6* mutations are associated with immunodeficiencies and *SHP1* mutations cause inflammatory phenotypes and autoimmune defects. Control of *S. typhimurium* and other infections in zebrafish is impaired both under conditions of a reduced or hyper-induced immune response, indicating the importance of highly balanced regulatory mechanisms (van der Vaart et al., 2013; Kanwal et al., 2013). Micro-RNAs (miRNAs), including members of the miR-146 family, have been implicated in fine-tuning of the mammalian innate immune response and, in zebrafish embryos, miR-146 is induced by *S. typhimurium* in a Myd88-Traf6-dependent manner. Although no major effects on known targets of the Myd88-Traf6 pathway were observed, apolipoprotein-mediated lipid transport emerged as a newly identified infection-inducible pathway under control of this miRNA family (Ordas et al., 2013).

The signals involved in recruitment of phagocytes to local infection remain to be elucidated. Chemokines, such as Cxcl8 (Il8) and the orphan ligand Cxcl-c1c, are highly induced rapidly upon *S. typhimurium* infection (Stockhammer et al., 2009). Using other bacterial infection models, the function of the CXCL8-CXCR2 signaling axis in neutrophil recruitment has been shown to be conserved in zebrafish (Sarris et al., 2012; Deng et al., 2013). Local *S. typhimurium* infection has also been shown to induce the recruitment of macrophages via the chemokine receptor Cxcr3.2, one of the zebrafish orthologs of human CXCR3 (Zakrzewska et al., 2010). The ligand association of Cxcr3.2 remains to be established.

In addition to phagocyte recruitment, localized infection also triggers emergency-driven hematopoiesis (Hall et al., 2012). Early neutropenia is a frequent outcome of *S. typhimurium* hindbrain infection in zebrafish embryos, and this is compensated for by increased granulopoiesis. The activity of iNOS (and thus the production of the pleiotropic mediator nitric oxide), was found to be necessary to stimulate the expansion and proliferation of HSPCs in response to infection-dependent neutropenia. The induction of iNOS is dependent on expression of the transcription factor C/ebpβ (CCAAT enhancer-binding protein β) in HSPCs. This is suggested to be an effect of elevated circulating levels of Gcsf (Granulocyte colony stimulating factor), produced by activated macrophages at the infection site (Hall et al., 2012).

The *S. typhimurium* infection model has recently led to new insight into the connection between infection control and host cell metabolic modulation (Hall et al., 2013). Profound adaptations in glucose and lipid metabolism occur within infected immune cells. For example, in response to stimulation by *Salmonella* pathogenic factors, macrophages increase their uptake of lipids to fuel ROS production (Hall et al., 2013). In line with this, *S. typhimurium* infection induces the expression of the mitochondria-associated enzyme Irg1 (Immunoresponsive gene 1) within infected zebrafish macrophages. This protein directs the catabolism of fatty acids to sustain mitochondrial oxidative phosphorylation and in turn leads to the production of mitochondrial ROS, contributing to intracellular degradation of phagocytosed bacteria. Irg1 holds promise as a new therapeutic target at the interface of inflammation and metabolism (Hall et al., 2013) (Table 1).

### Burkholderia cenocepacia

The *B. cepacia* complex (*Bcc*) is represented by several closely related Gram-negative species that are able to survive freely in the environment or replicate within different hosts, including amoebae, invertebrates, vertebrates and plants (Mahenthiralingam et al., 2008). In humans, opportunistic infection by *Bcc* frequently occurs in cystic fibrosis or immunocompromised individuals and represents a recurrent cause of fatal complications (Saiman and Siegel, 2004). The capability to survive and infect a wide range of hosts suggests that *Bcc* species are highly adaptable to different niches and produce multiple virulence factors; however, the complex mechanisms of host-pathogen interactions underlying infections with *B. cenocepacia* and other *Bcc* strains remain largely unknown. In particular, it has been difficult to establish conclusively whether *B. cenocepacia* can survive intracellularly. Visualizing infection in zebrafish embryos helped to answer this key question, by demonstrating the ability of this pathogen to survive within macrophages (Fig. 3C). Following the creation of an intramacrophage replication niche, the bacterial infection disseminates by non-lytic expulsion from infected cells, induces immune cell aggregations and ultimately causes fatal systemic bacteremia (Vergunst et al., 2010).

The zebrafish model system has also provided a valuable contribution to the investigation of the *in vivo* relevance of several *B. cenocepacia* virulence factors. A quorum-sensing-deficient cepR strain was shown to be strongly attenuated, indicated by a reduced ability to replicate intracellularly and to disseminate efficiently from infected macrophages. Furthermore, differences in virulence were observed between strains from a panel of clinical isolates (Vergunst et al., 2010). Loss of the third replicon, pC3 (a non-essential megaplasmid associated with several virulence determinants), results in highly attenuated infection in multiple hosts, including zebrafish embryos (Agnoli et al., 2012; Agnoli et al., 2014). Mutants in pC3 are not able to grow significantly *in vivo*, but are not eradicated, suggesting that the pC3-linked virulence factors are dispensable for intramacrophage survival (Agnoli et al., 2012). A function in adaptation to a wide range of environments, rather than a direct role in modulating intracellular growth, might explain the prevalence of the pC3 replicon among *Bcc* isolates (Agnoli et al., 2014).

Together, these studies demonstrate the usefulness of the zebrafish model for analysis of *B. cenocepacia* mechanisms of intracellular survival and virulence.

### Staphylococcus aureus

*S. aureus* is the causative agent of a wide range of infectious pathologies such as sty, pneumonia, endocarditis, osteomyelitis and septicemia (Lowy, 1998), which remain important causes of morbidity and of complications in hospitalized patients. Although not a natural pathogen of zebrafish, both embryos (Prajsnar et al., 2008) and adults (Lin et al., 2007) exhibit clear acute symptoms when infected with this Gram-positive pathogen, providing a useful model for bacteremia (Fig. 3D).

*S. aureus* has long been considered an extracellular pathogen, but there is accumulating evidence that it can also survive and replicate in phagocytes (Rigby and DeLeo, 2012). The zebrafish embryo model has contributed significantly to our understanding of the nature and relevance of the intracellular phase in the life cycle of this pathogen (Prajsnar et al., 2008; Prajsnar et al., 2012). Live imaging showed that, upon intravenous infection, *S. aureus* is completely phagocytosed by macrophages and neutrophils (Prajsnar et al., 2008; Li and Hu, 2012). Although some embryos clear the infection in a phagocyte-dependent manner, other embryos develop overwhelming infection, indicating that the bacteria can subvert the phagocyte-killing mechanisms (Prajsnar et al., 2012).

When larvae are co-infected with two isogenic, but differently labeled, *S. aureus* clones, the infection evolves by forming focal lesions that are predominantly monoclonal and, during the course of overwhelming infection, the ratio between the original strains is often skewed towards one predominating strain (Prajsnar et al., 2012). This phenomenon is fully dependent on phagocyte activity. These data suggest that most of the phagocytes are able to clear the infection but a population of phagocytes provides an intracellular protective niche in which some bacteria gain the ability to replicate and resist, to ultimately be released and disseminate. Consistent with this, co-infection in a murine sepsis model resulted in kidney abscesses that contained predominantly one strain of *S. aureus* and thus were likely founded by single bacteria (Holtfreter et al., 2013). The relevance of this work for clinical treatments is underscored by a recent study showing that the use of sub-curative antibiotic doses can support the preferential expansion of antibiotic-resistant bacteria during a mixed infection (McVicker et al., 2014). Selective ablation of macrophages or neutrophils in the zebrafish model has revealed that neutrophils are most likely to form the privileged niche responsible for disseminated infection of *S. aureus* (Prajsnar et al., 2012). Interesting remaining questions include elucidation of the mechanisms by which some bacteria from the initial inoculum are able to avoid being killed by neutrophils, and determination of whether *S. aureus* can also resist macrophages *in vivo*, as suggested by human cell culture studies (Kubica et al., 2008).

### Shigella flexneri

*S. flexneri*, a human-adapted *Escherichia coli* species, is a causative agent of diarrhea and dysentery in humans, generally deriving from orofecal contaminations. Like other enterobacteria, it mostly affects the digestive tract; however, in advanced infectious stages, it can lead to bacteremia and systemic sepsis. In the early phase of infection, the pathogen can interact with membranes of host cells, inject virulence determinants, and induce ruffling and internalization (Ogawa et al., 2008). In this actively induced ingestion mechanism resides its capability to establish intracellular infection in non-phagocytic cells, such as epithelial cells associated with the digestive tract. Once it is internalized, it can escape from phagosomes and survive freely in the cytosol. Subsequently, the pathogen can spread through intestinal epithelial cells by actin-based motility (Fig. 3E). Microfold cells allow *Shigella* to transverse the intestinal epithelium, where they encounter macrophages. Death of infected macrophages and subsequent destabilization of the epithelium due to inflammation allows more *Shigella* to infiltrate the tissue and invade epithelial cells through the basal membrane. Survival and replication in macrophages, eventually followed by macrophage pyroptosis, is fundamental to allowing dissemination and extensive colonization of the intestinal epithelium (Ashida et al., 2011).

Recently, a zebrafish model for *S. flexneri* was established, and this has been used to show that intravenously administrated bacteria can survive and replicate both in macrophages and in non-immune cells (Mostowy et al., 2013). The pathogenicity of *Shigella* is highly dependent on the presence of T3SS virulence factors, and avirulent strains can be successfully combated by the zebrafish innate immune system and are not able to induce phagocytosis in non-phagocytic cells. Live imaging shows that replication of *S. flexneri* in macrophages ultimately induces cell death, whereas bacteria are more efficiently contained and degraded within neutrophils. Neutrophils also act as scavengers, eliminating infected dead cells. Macrophages are not able to retain the infection within vacuoles and bacteria spread into the cytosol, where they can colocalize with actin and septin cages (Fig. 3E).

In mammalian cultured cells, cytosolic *S. flexneri* can be targeted for autophagy via both ubiquitin-dependent and -independent pathways, and, as a counteractive mechanism, the bacteria can secrete virulence factors to escape autophagy (Ogawa et al., 2005; Mostowy et al., 2011). Colocalization with the autophagy marker Lc3 followed by electron microscopic analysis in the zebrafish model confirmed that autophagy targeting is associated with entrapment of *S. flexneri* in septin cage-like structures (Mostowy et al., 2010; Mostowy et al., 2013). Reduction of autophagy, via knockdown of the autophagy-related receptor p62, increases the infection burden of zebrafish larvae and this effect is specific only for the T3SS-positive strain that is able to escape into the cytosol (Mostowy et al., 2013). These data support the hypothesis that antibacterial protection provided by efficient autophagic machinery is essential to properly counteract *S. flexneri* infection. The ability to monitor *S. flexneri* infection in a transparent zebrafish host provides new possibilities to assess the relevance of autophagy *in vivo* in immune and non-immune cells, and to develop new strategies for anti-bacterial therapies targeting this process (Table 1).

### Candida albicans

*C. albicans* is an opportunistic dimorphic fungus that grows in yeast and hyphal forms (Gow et al., 2011). Most of the human population carries *C. albicans* as a harmless constituent of the epidermal, mucosal and intestinal flora. However, uncontrolled systemic candidiasis and fungal growth on the mucosal surfaces can cause severe and life-threatening infectious complications, particularly in immunocompromised individuals. In zebrafish embryos, as in humans, *C. albicans* can be phagocytosed by both neutrophils and macrophages (Chao et al., 2010; Brothers et al., 2011). Live observations reveal that, in a non-compromised zebrafish host, this intracellular localization leads to a transitory standoff phase in which the yeast form survives and replicates, but does not germinate or lyse the host cell (Fig. 3F). Subsequently, the fungi switch to the more virulent hyphal form and proliferate exuberantly in individuals that fail to contain the infection, whereas they revert to the yeast form in most surviving embryos (Brothers et al., 2011). Intracellular yeast forms are unable to undergo the yeast-to-hyphal transition, even under conditions of impaired oxidative-stress response, in contrast with previous *in vitro* data in which filamentous growth was observed within cultured macrophages (Brothers et al., 2013). Therefore, macrophages apparently have an enhanced ability to control infection in the *in vivo* environment.

Although germination was shown to be independent of the phagocyte-specific NADPH oxidase (PHOX), this enzyme was found to be essential to produce an efficient oxidative-stress response against *C. albicans* and to control filamentous growth (Brothers et al., 2011; Brothers et al., 2013). Previously, the limitation of fungal growth was ascribed mainly to direct fungal destruction by ROS; however, imaging in zebrafish revealed a non-canonical role for PHOX and for the epithelial dual NADPH oxidase (DUOX) in recruitment of phagocytes to *C. albicans* infection sites. Therefore, impaired phagocyte recruitment to invading *Candida* under conditions of NADPH oxidase deficiency seems to be the cause of the overall reduction in containment of the infection and, consequently, of massive extracellular hyphal growth. Although localized infection with wild-type *C. albicans* is unable to induce chemoattraction under conditions of NADPH oxidase deficiency, infection with a yeast-locked mutant strain (*edt1Δ/Δ*) can be efficiently counteracted by phagocyte recruitment and internalization, even in pan-NADPH-oxidase-depleted conditions. This suggests that the hyphal transition (or another *edt1*-associated program) is also able to attenuate ROS-independent phagocyte recruitment, thus explaining the relevance of host ROS-driven chemoattraction mechanisms to counteract *C. albicans* infection (Brothers et al., 2013).

## Concluding remarks

The study of intracellular pathogens in zebrafish macrophages has led to new mechanistic insights that are inspiring novel host-directed therapeutic strategies (Table 1). The real-time imaging possibilities in zebrafish will also be very useful for elucidating the mechanisms underlying macrophage migration processes, as has already been demonstrated by the study of neutrophils in the larval system (Sarris et al., 2012; Henry et al., 2013; Shelef et al., 2013). A question that is very relevant both for infectious diseases and for cancer biology concerns the presence of different pro- and anti-inflammatory macrophage subtypes in zebrafish. Classically activated (M1) and alternatively activated (M2) macrophages, resembling the phenotypes of mammalian macrophages, have been identified in different fish species (Forlenza et al., 2011). That different macrophage subtypes might already be present in early zebrafish larvae has been suggested, but this remains to be further investigated (Feng et al., 2010). The early larval stages, which are optimally suited for imaging and for genetic and pharmacological interventions, can give much information on the intracellular survival mechanisms of pathogens, as demonstrated by the studies discussed herein. The early larval stages are also very useful for studying the response of microglia to brain injuries or infection, contributing to a deeper understanding of the role of these specialized macrophages in neurodegenerative diseases (Sieger et al., 2012; Sieger and Peri, 2013). Studying the antigen-presentation function of macrophages and DCs at later developmental stages is becoming increasingly feasible owing to advances in technologies for generating stable mutant lines (Clark et al., 2011; Blackburn et al., 2013; Kettleborough et al., 2013). Dynamic interactions between macrophages and neutrophils that are emerging from recent studies in zebrafish are of considerable interest for further study (Ellett et al., 2011; Yang et al., 2012; Elks et al., 2013). The use of the zebrafish model has already provided insights into the *in vivo* relevance of intracellular defense mechanisms such as ROS and RNS production and autophagy. We expect that further use of this powerful model will continue to make important contributions towards the understanding of innate immunity and of the virulence strategies that pathogens use to subvert innate host defenses.

## Supplementary Material

Supplementary Material

## References

[b1-0070785] Abdallah A. M. Gey van Pittius N. C. Champion P. A. Cox J. Luirink J. Vandenbroucke-Grauls C. M. Appelmelk B. J. Bitter W. (2007). Type VII secretion – mycobacteria show the way. Nat. Rev. Microbiol. 5, 883–891 17922044 10.1038/nrmicro1773

[b2-0070785] Adams K. N. Takaki K. Connolly L. E. Wiedenhoft H. Winglee K. Humbert O. Edelstein P. H. Cosma C. L. Ramakrishnan L. (2011). Drug tolerance in replicating mycobacteria mediated by a macrophage-induced efflux mechanism. Cell 145, 39–53 21376383 10.1016/j.cell.2011.02.022 PMC3117281

[b3-0070785] Adams K. N. Szumowski J. D. Ramakrishnan L. (2014). Verapamil, and its metabolite norverapamil, inhibit macrophage-induced, bacterial efflux pump-mediated tolerance to multiple anti-tubercular drugs. J. Infect. Dis. [Epub ahead of print] 10.1093/infdis/jiu095 PMC4110457 24532601

[b4-0070785] Agnoli K. Schwager S. Uehlinger S. Vergunst A. Viteri D. F. Nguyen D. T. Sokol P. A. Carlier A. Eberl L. (2012). Exposing the third chromosome of Burkholderia cepacia complex strains as a virulence plasmid. Mol. Microbiol. 83, 362–378 22171913 10.1111/j.1365-2958.2011.07937.x

[b5-0070785] Agnoli K. Frauenknecht C. Freitag R. Schwager S. Jenul C. Vergunst A. Carlier A. Eberl L. (2014). The third replicon of members of the Burkholderia cepacia Complex, plasmid pC3, plays a role in stress tolerance. Appl. Environ. Microbiol. 80, 1340–1348 24334662 10.1128/AEM.03330-13 PMC3911052

[b6-0070785] Ashida H. Ogawa M. Kim M. Suzuki S. Sanada T. Punginelli C. Mimuro H. Sasakawa C. (2011). Shigella deploy multiple countermeasures against host innate immune responses. Curr. Opin. Microbiol. 14, 16–23 20934372 10.1016/j.mib.2010.08.014

[b7-0070785] Baxt L. A. Garza-Mayers A. C. Goldberg M. B. (2013). Bacterial subversion of host innate immune pathways. Science 340, 697–701 23661751 10.1126/science.1235771

[b8-0070785] Benard E. L. van der Sar A. M. Ellett F. Lieschke G. J. Spaink H. P. Meijer A. H. (2012). Infection of zebrafish embryos with intracellular bacterial pathogens. J. Vis. Exp. 61, 3781 22453760 10.3791/3781 PMC3415172

[b9-0070785] Bernut A. Herrmann J. L. Kissa K. Dubremetz J. F. Gaillard J. L. Lutfalla G. Kremer L. (2014). Mycobacterium abscessus cording prevents phagocytosis and promotes abscess formation. Proc. Natl. Acad. Sci. USA 111, E943–E952 24567393 10.1073/pnas.1321390111 PMC3956181

[b10-0070785] Bertin J. Nir W. J. Fischer C. M. Tayber O. V. Errada P. R. Grant J. R. Keilty J. J. Gosselin M. L. Robison K. E. Wong G. H. (1999). Human CARD4 protein is a novel CED-4/Apaf-1 cell death family member that activates NF-kappaB. J. Biol. Chem. 274, 12955–12958 10224040 10.1074/jbc.274.19.12955

[b11-0070785] Bertrand J. Y. Kim A. D. Violette E. P. Stachura D. L. Cisson J. L. Traver D. (2007). Definitive hematopoiesis initiates through a committed erythromyeloid progenitor in the zebrafish embryo. Development 134, 4147–4156 17959717 10.1242/dev.012385 PMC2735398

[b12-0070785] Bertrand J. Y. Chi N. C. Santoso B. Teng S. Stainier D. Y. Traver D. (2010). Haematopoietic stem cells derive directly from aortic endothelium during development. Nature 464, 108–111 20154733 10.1038/nature08738 PMC2858358

[b13-0070785] Bilan D. S. Pase L. Joosen L. Gorokhovatsky A. Y. Ermakova Y. G. Gadella T. W. Grabher C. Schultz C. Lukyanov S. Belousov V. V. (2013). HyPer-3: a genetically encoded H(2)O(2) probe with improved performance for ratiometric and fluorescence lifetime imaging. ACS Chem. Biol. 8, 535–542 23256573 10.1021/cb300625g

[b14-0070785] Blackburn P. R. Campbell J. M. Clark K. J. Ekker S. C. (2013). The CRISPR system – keeping zebrafish gene targeting fresh. Zebrafish 10, 116–118 23536990 10.1089/zeb.2013.9999 PMC3629780

[b15-0070785] Boisset J. C. van Cappellen W. Andrieu-Soler C. Galjart N. Dzierzak E. Robin C. (2010). In vivo imaging of haematopoietic cells emerging from the mouse aortic endothelium. Nature 464, 116–120 20154729 10.1038/nature08764

[b16-0070785] Brothers K. M. Newman Z. R. Wheeler R. T. (2011). Live imaging of disseminated candidiasis in zebrafish reveals role of phagocyte oxidase in limiting filamentous growth. Eukaryot. Cell 10, 932–944 21551247 10.1128/EC.05005-11 PMC3147414

[b17-0070785] Brothers K. M. Gratacap R. L. Barker S. E. Newman Z. R. Norum A. Wheeler R. T. (2013). NADPH oxidase-driven phagocyte recruitment controls Candida albicans filamentous growth and prevents mortality. PLoS Pathog. 9, e1003634 24098114 10.1371/journal.ppat.1003634 PMC3789746

[b18-0070785] Cambier C. J. Takaki K. K. Larson R. P. Hernandez R. E. Tobin D. M. Urdahl K. B. Cosma C. L. Ramakrishnan L. (2014). Mycobacteria manipulate macrophage recruitment through coordinated use of membrane lipids. Nature 505, 218–222 24336213 10.1038/nature12799 PMC3961847

[b19-0070785] Carvalho R. de Sonneville J. Stockhammer O. W. Savage N. D. Veneman W. J. Ottenhoff T. H. Dirks R. P. Meijer A. H. Spaink H. P. (2011). A high-throughput screen for tuberculosis progression. PLoS ONE 6, e16779 21390204 10.1371/journal.pone.0016779 PMC3040195

[b20-0070785] Chao C. C. Hsu P. C. Jen C. F. Chen I. H. Wang C. H. Chan H. C. Tsai P. W. Tung K. C. Wang C. H. Lan C. Y. (2010). Zebrafish as a model host for Candida albicans infection. Infect. Immun. 78, 2512–2521 20308295 10.1128/IAI.01293-09 PMC2876552

[b21-0070785] Cheung C. Y. Webb S. E. Love D. R. Miller A. L. (2011). Visualization, characterization and modulation of calcium signaling during the development of slow muscle cells in intact zebrafish embryos. Int. J. Dev. Biol. 55, 153–174 21553383 10.1387/ijdb.103160cc

[b22-0070785] Clark K. J. Voytas D. F. Ekker S. C. (2011). A TALE of two nucleases: gene targeting for the masses? Zebrafish 8, 147–149 21929364 10.1089/zeb.2011.9993 PMC3174730

[b23-0070785] Clay H. Volkman H. E. Ramakrishnan L. (2008). Tumor necrosis factor signaling mediates resistance to mycobacteria by inhibiting bacterial growth and macrophage death. Immunity 29, 283–294 18691913 10.1016/j.immuni.2008.06.011 PMC3136176

[b24-0070785] Colucci-Guyon E. Tinevez J. Y. Renshaw S. A. Herbomel P. (2011). Strategies of professional phagocytes in vivo: unlike macrophages, neutrophils engulf only surface-associated microbes. J. Cell Sci. 124, 3053–3059 21868367 10.1242/jcs.082792

[b25-0070785] Cotton M. Claing A. (2009). G protein-coupled receptors stimulation and the control of cell migration. Cell. Signal. 21, 1045–1053 19249352 10.1016/j.cellsig.2009.02.008

[b26-0070785] Cronan M. R. Tobin D. M. (2014). Fit for consumption: zebrafish as a model for tuberculosis. Dis. Model. Mech. 7, 777–784 10.1242/dmm.016089 PMC4073268 24973748

[b27-0070785] Davis J. M. Ramakrishnan L. (2009). The role of the granuloma in expansion and dissemination of early tuberculous infection. Cell 136, 37–49 19135887 10.1016/j.cell.2008.11.014 PMC3134310

[b28-0070785] Davis J. M. Clay H. Lewis J. L. Ghori N. Herbomel P. Ramakrishnan L. (2002). Real-time visualization of mycobacterium-macrophage interactions leading to initiation of granuloma formation in zebrafish embryos. Immunity 17, 693–702 12479816 10.1016/s1074-7613(02)00475-2

[b29-0070785] Deng Q. Sarris M. Bennin D. A. Green J. M. Herbomel P. Huttenlocher A. (2013). Localized bacterial infection induces systemic activation of neutrophils through Cxcr2 signaling in zebrafish. J. Leukoc. Biol. 93, 761–769 23475575 10.1189/jlb.1012534 PMC4050646

[b30-0070785] Deretic V. Saitoh T. Akira S. (2013). Autophagy in infection, inflammation and immunity. Nat. Rev. Immunol. 13, 722–737 24064518 10.1038/nri3532 PMC5340150

[b31-0070785] Duclos S. Desjardins M. (2000). Subversion of a young phagosome: the survival strategies of intracellular pathogens. Cell. Microbiol. 2, 365–377 11207592 10.1046/j.1462-5822.2000.00066.x

[b32-0070785] Dunlap N. E. Benjamin W. H. Jr Berry A. K. Eldridge J. H. Briles D. E. (1992). A ‘safe-site’ for Salmonella typhimurium is within splenic polymorphonuclear cells. Microb. Pathog. 13, 181–190 1291841 10.1016/0882-4010(92)90019-k

[b33-0070785] El-Gayar S. Thüring-Nahler H. Pfeilschifter J. Röllinghoff M. Bogdan C. (2003). Translational control of inducible nitric oxide synthase by IL-13 and arginine availability in inflammatory macrophages. J. Immunol. 171, 4561–4568 14568929 10.4049/jimmunol.171.9.4561

[b34-0070785] Elks P. M. Brizee S. van der Vaart M. Walmsley S. R. van Eeden F. J. Renshaw S. A. Meijer A. H. (2013). Hypoxia inducible factor signaling modulates susceptibility to mycobacterial infection via a nitric oxide dependent mechanism. PLoS Pathog. 9, e1003789 24367256 10.1371/journal.ppat.1003789 PMC3868520

[b35-0070785] Ellett F. Pase L. Hayman J. W. Andrianopoulos A. Lieschke G. J. (2011). mpeg1 promoter transgenes direct macrophage-lineage expression in zebrafish. Blood 117, e49–e56 21084707 10.1182/blood-2010-10-314120 PMC3056479

[b36-0070785] Elomaa O. Kangas M. Sahlberg C. Tuukkanen J. Sormunen R. Liakka A. Thesleff I. Kraal G. Tryggvason K. (1995). Cloning of a novel bacteria-binding receptor structurally related to scavenger receptors and expressed in a subset of macrophages. Cell 80, 603–609 7867067 10.1016/0092-8674(95)90514-6

[b37-0070785] Fàbrega A. Vila J. (2013). Salmonella enterica serovar Typhimurium skills to succeed in the host: virulence and regulation. Clin. Microbiol. Rev. 26, 308–341 23554419 10.1128/CMR.00066-12 PMC3623383

[b38-0070785] Feng Y. Santoriello C. Mione M. Hurlstone A. Martin P. (2010). Live imaging of innate immune cell sensing of transformed cells in zebrafish larvae: parallels between tumor initiation and wound inflammation. PLoS Biol. 8, e1000562 21179501 10.1371/journal.pbio.1000562 PMC3001901

[b39-0070785] Forlenza M. Fink I. R. Raes G. Wiegertjes G. F. (2011). Heterogeneity of macrophage activation in fish. Dev. Comp. Immunol. 35, 1246–1255 21414343 10.1016/j.dci.2011.03.008

[b40-0070785] Gengenbacher M. Kaufmann S. H. (2012). Mycobacterium tuberculosis: success through dormancy. FEMS Microbiol. Rev. 36, 514–532 22320122 10.1111/j.1574-6976.2012.00331.x PMC3319523

[b41-0070785] Gow N. A. van de Veerdonk F. L. Brown A. J. Netea M. G. (2011). Candida albicans morphogenesis and host defence: discriminating invasion from colonization. Nat. Rev. Microbiol. 10, 112–122 22158429 10.1038/nrmicro2711 PMC3624162

[b42-0070785] Gray C. Loynes C. A. Whyte M. K. Crossman D. C. Renshaw S. A. Chico T. J. (2011). Simultaneous intravital imaging of macrophage and neutrophil behaviour during inflammation using a novel transgenic zebrafish. Thromb. Haemost. 105, 811–819 21225092 10.1160/TH10-08-0525

[b43-0070785] Hall C. Flores M. V. Chien A. Davidson A. Crosier K. Crosier P. (2009). Transgenic zebrafish reporter lines reveal conserved Toll-like receptor signaling potential in embryonic myeloid leukocytes and adult immune cell lineages. J. Leukoc. Biol. 85, 751–765 19218482 10.1189/jlb.0708405

[b44-0070785] Hall C. J. Flores M. V. Oehlers S. H. Sanderson L. E. Lam E. Y. Crosier K. E. Crosier P. S. (2012). Infection-responsive expansion of the hematopoietic stem and progenitor cell compartment in zebrafish is dependent upon inducible nitric oxide. Cell Stem Cell 10, 198–209 22305569 10.1016/j.stem.2012.01.007

[b45-0070785] Hall C. J. Boyle R. H. Astin J. W. Flores M. V. Oehlers S. H. Sanderson L. E. Ellett F. Lieschke G. J. Crosier K. E. Crosier P. S. (2013). Immunoresponsive gene 1 augments bactericidal activity of macrophage-lineage cells by regulating β-oxidation-dependent mitochondrial ROS production. Cell Metab. 18, 265–278 23931757 10.1016/j.cmet.2013.06.018

[b46-0070785] Henry K. M. Loynes C. A. Whyte M. K. Renshaw S. A. (2013). Zebrafish as a model for the study of neutrophil biology. J. Leukoc. Biol. 94, 633–642 23463724 10.1189/jlb.1112594

[b47-0070785] Herbomel P. Levraud J. P. (2005). Imaging early macrophage differentiation, migration, and behaviors in live zebrafish embryos. Methods Mol. Med. 105, 199–214 15492397 10.1385/1-59259-826-9:199

[b48-0070785] Herbomel P. Thisse B. Thisse C. (1999). Ontogeny and behaviour of early macrophages in the zebrafish embryo. Development 126, 3735–3745 10433904 10.1242/dev.126.17.3735

[b49-0070785] Herbomel P. Thisse B. Thisse C. (2001). Zebrafish early macrophages colonize cephalic mesenchyme and developing brain, retina, and epidermis through a M-CSF receptor-dependent invasive process. Dev. Biol. 238, 274–288 11784010 10.1006/dbio.2001.0393

[b50-0070785] Hilbi H. Moss J. E. Hersh D. Chen Y. Arondel J. Banerjee S. Flavell R. A. Yuan J. Sansonetti P. J. Zychlinsky A. (1998). Shigella-induced apoptosis is dependent on caspase-1 which binds to IpaB. J. Biol. Chem. 273, 32895–32900 9830039 10.1074/jbc.273.49.32895

[b51-0070785] Holtfreter S. Radcliff F. J. Grumann D. Read H. Johnson S. Monecke S. Ritchie S. Clow F. Goerke C. Bröker B. M. (2013). Characterization of a mouse-adapted Staphylococcus aureus strain. PLoS ONE 8, e71142 24023720 10.1371/journal.pone.0071142 PMC3759423

[b52-0070785] Inohara N. Koseki T. del Peso L. Hu Y. Yee C. Chen S. Carrio R. Merino J. Liu D. Ni J. (1999). Nod1, an Apaf-1-like activator of caspase-9 and nuclear factor-kappaB. J. Biol. Chem. 274, 14560–14567 10329646 10.1074/jbc.274.21.14560

[b53-0070785] Jagannathan-Bogdan M. Zon L. I. (2013). Hematopoiesis. Development 140, 2463–2467 23715539 10.1242/dev.083147 PMC3666375

[b54-0070785] Kanwal Z. Zakrzewska A. den Hertog J. Spaink H. P. Schaaf M. J. Meijer A. H. (2013). Deficiency in hematopoietic phosphatase ptpn6/Shp1 hyperactivates the innate immune system and impairs control of bacterial infections in zebrafish embryos. J. Immunol. 190, 1631–1645 23335748 10.4049/jimmunol.1200551

[b55-0070785] Kettleborough R. N. Busch-Nentwich E. M. Harvey S. A. Dooley C. M. de Bruijn E. van Eeden F. Sealy I. White R. J. Herd C. Nijman I. J. (2013). A systematic genome-wide analysis of zebrafish protein-coding gene function. Nature 496, 494–497 23594742 10.1038/nature11992 PMC3743023

[b56-0070785] Kissa K. Herbomel P. (2010). Blood stem cells emerge from aortic endothelium by a novel type of cell transition. Nature 464, 112–115 20154732 10.1038/nature08761

[b57-0070785] Kubica M. Guzik K. Koziel J. Zarebski M. Richter W. Gajkowska B. Golda A. Maciag-Gudowska A. Brix K. Shaw L. (2008). A potential new pathway for Staphylococcus aureus dissemination: the silent survival of S. aureus phagocytosed by human monocyte-derived macrophages. PLoS ONE 3, e1409 18183290 10.1371/journal.pone.0001409 PMC2169301

[b58-0070785] Lam S. H. Chua H. L. Gong Z. Lam T. J. Sin Y. M. (2004). Development and maturation of the immune system in zebrafish, Danio rerio: a gene expression profiling, in situ hybridization and immunological study. Dev. Comp. Immunol. 28, 9–28 12962979 10.1016/s0145-305x(03)00103-4

[b59-0070785] Li Y. J. Hu B. (2012). Establishment of multi-site infection model in zebrafish larvae for studying Staphylococcus aureus infectious disease. J. Genet. Genomics 39, 521–534 23021551 10.1016/j.jgg.2012.07.006

[b60-0070785] Li L. Jin H. Xu J. Shi Y. Wen Z. (2011). Irf8 regulates macrophage versus neutrophil fate during zebrafish primitive myelopoiesis. Blood 117, 1359–1369 21079149 10.1182/blood-2010-06-290700

[b61-0070785] Lin B. Chen S. Cao Z. Lin Y. Mo D. Zhang H. Gu J. Dong M. Liu Z. Xu A. (2007). Acute phase response in zebrafish upon Aeromonas salmonicida and Staphylococcus aureus infection: striking similarities and obvious differences with mammals. Mol. Immunol. 44, 295–301 16630661 10.1016/j.molimm.2006.03.001

[b62-0070785] Lin P. L. Rodgers M. Smith L. Bigbee M. Myers A. Bigbee C. Chiosea I. Capuano S. V. Fuhrman C. Klein E. (2009). Quantitative comparison of active and latent tuberculosis in the cynomolgus macaque model. Infect. Immun. 77, 4631–4642 19620341 10.1128/IAI.00592-09 PMC2747916

[b63-0070785] Lowy F. D. (1998). Staphylococcus aureus infections. N. Engl. J. Med. 339, 520–532 9709046 10.1056/NEJM199808203390806

[b64-0070785] Mahenthiralingam E. Baldwin A. Dowson C. G. (2008). Burkholderia cepacia complex bacteria: opportunistic pathogens with important natural biology. J. Appl. Microbiol. 104, 1539–1551 18217926 10.1111/j.1365-2672.2007.03706.x

[b65-0070785] Martinon F. Burns K. Tschopp J. (2002). The inflammasome: a molecular platform triggering activation of inflammatory caspases and processing of proIL-beta. Mol. Cell 10, 417–426 12191486 10.1016/s1097-2765(02)00599-3

[b66-0070785] Masaki T. Qu J. Cholewa-Waclaw J. Burr K. Raaum R. Rambukkana A. (2013). Reprogramming adult Schwann cells to stem cell-like cells by leprosy bacilli promotes dissemination of infection. Cell 152, 51–67 23332746 10.1016/j.cell.2012.12.014 PMC4314110

[b67-0070785] McVicker G. Prajsnar T. K. Williams A. Wagner N. L. Boots M. Renshaw S. A. Foster S. J. (2014). Clonal expansion during Staphylococcus aureus infection dynamics reveals the effect of antibiotic intervention. PLoS Pathog. 10, e1003959 24586163 10.1371/journal.ppat.1003959 PMC3937288

[b68-0070785] Meijer A. H. van der Sar A. M. Cunha C. Lamers G. E. Laplante M. A. Kikuta H. Bitter W. Becker T. S. Spaink H. P. (2008). Identification and real-time imaging of a myc-expressing neutrophil population involved in inflammation and mycobacterial granuloma formation in zebrafish. Dev. Comp. Immunol. 32, 36–49 17553562 10.1016/j.dci.2007.04.003

[b69-0070785] Meijer A. H. van der Vaart M. Spaink H. P. (2014). Real-time imaging and genetic dissection of host-microbe interactions in zebrafish. Cell. Microbiol. 16, 39–49 PubMed 24188444 10.1111/cmi.12236

[b70-0070785] Minakami R. Sumimotoa H. (2006). Phagocytosis-coupled activation of the superoxide-producing phagocyte oxidase, a member of the NADPH oxidase (nox) family. Int. J. Hematol. 84, 193–198 17050190 10.1532/IJH97.06133

[b71-0070785] Mostowy S. Bonazzi M. Hamon M. A. Tham T. N. Mallet A. Lelek M. Gouin E. Demangel C. Brosch R. Zimmer C. (2010). Entrapment of intracytosolic bacteria by septin cage-like structures. Cell Host Microbe 8, 433–444 21075354 10.1016/j.chom.2010.10.009

[b72-0070785] Mostowy S. Sancho-Shimizu V. Hamon M. A. Simeone R. Brosch R. Johansen T. Cossart P. (2011). p62 and NDP52 proteins target intracytosolic Shigella and Listeria to different autophagy pathways. J. Biol. Chem. 286, 26987–26995 21646350 10.1074/jbc.M111.223610 PMC3143657

[b73-0070785] Mostowy S. Boucontet L. Mazon Moya M. J. Sirianni A. Boudinot P. Hollinshead M. Cossart P. Herbomel P. Levraud J. P. Colucci-Guyon E. (2013). The zebrafish as a new model for the in vivo study of Shigella flexneri interaction with phagocytes and bacterial autophagy. PLoS Pathog. 9, e1003588 24039575 10.1371/journal.ppat.1003588 PMC3764221

[b74-0070785] Murayama E. Kissa K. Zapata A. Mordelet E. Briolat V. Lin H. F. Handin R. I. Herbomel P. (2006). Tracing hematopoietic precursor migration to successive hematopoietic organs during zebrafish development. Immunity 25, 963–975 17157041 10.1016/j.immuni.2006.10.015

[b75-0070785] O’Neill L. A. Golenbock D. Bowie A. G. (2013). The history of Toll-like receptors – redefining innate immunity. Nat. Rev. Immunol. 13, 453–460 23681101 10.1038/nri3446

[b76-0070785] Ogawa M. Yoshimori T. Suzuki T. Sagara H. Mizushima N. Sasakawa C. (2005). Escape of intracellular Shigella from autophagy. Science 307, 727–731 15576571 10.1126/science.1106036

[b77-0070785] Ogawa M. Handa Y. Ashida H. Suzuki M. Sasakawa C. (2008). The versatility of Shigella effectors. Nat. Rev. Microbiol. 6, 11–16 18059288 10.1038/nrmicro1814

[b78-0070785] Ordas A. Hegedus Z. Henkel C. V. Stockhammer O. W. Butler D. Jansen H. J. Racz P. Mink M. Spaink H. P. Meijer A. H. (2011). Deep sequencing of the innate immune transcriptomic response of zebrafish embryos to Salmonella infection. Fish Shellfish Immunol. 31, 716–724 20816807 10.1016/j.fsi.2010.08.022

[b79-0070785] Ordas A. Kanwal Z. Lindenberg V. Rougeot J. Mink M. Spaink H. P. Meijer A. H. (2013). MicroRNA-146 function in the innate immune transcriptome response of zebrafish embryos to Salmonella typhimurium infection. BMC Genomics 14, 696 24112639 10.1186/1471-2164-14-696 PMC3852110

[b80-0070785] Ottenhoff T. H. Kaufmann S. H. (2012). Vaccines against tuberculosis: where are we and where do we need to go? PLoS Pathog. 8, e1002607 22589713 10.1371/journal.ppat.1002607 PMC3349743

[b81-0070785] Page D. M. Wittamer V. Bertrand J. Y. Lewis K. L. Pratt D. N. Delgado N. Schale S. E. McGue C. Jacobsen B. H. Doty A. (2013). An evolutionarily conserved program of B-cell development and activation in zebrafish. Blood 122, e1–e11 23861249 10.1182/blood-2012-12-471029 PMC3750348

[b82-0070785] Parikka M. Hammarén M. M. Harjula S. K. Halfpenny N. J. Oksanen K. E. Lahtinen M. J. Pajula E. T. Iivanainen A. Pesu M. Rämet M. (2012). Mycobacterium marinum causes a latent infection that can be reactivated by gamma irradiation in adult zebrafish. PLoS Pathog. 8, e1002944 23028333 10.1371/journal.ppat.1002944 PMC3459992

[b83-0070785] Park Y. K. Bearson B. Bang S. H. Bang I. S. Foster J. W. (1996). Internal pH crisis, lysine decarboxylase and the acid tolerance response of Salmonella typhimurium. Mol. Microbiol. 20, 605–611 8736539 10.1046/j.1365-2958.1996.5441070.x

[b84-0070785] Peri F. Nüsslein-Volhard C. (2008). Live imaging of neuronal degradation by microglia reveals a role for v0-ATPase a1 in phagosomal fusion in vivo. Cell 133, 916–927 18510934 10.1016/j.cell.2008.04.037

[b85-0070785] Prajsnar T. K. Cunliffe V. T. Foster S. J. Renshaw S. A. (2008). A novel vertebrate model of Staphylococcus aureus infection reveals phagocyte-dependent resistance of zebrafish to non-host specialized pathogens. Cell. Microbiol. 10, 2312–2325 18715285 10.1111/j.1462-5822.2008.01213.x

[b86-0070785] Prajsnar T. K. Hamilton R. Garcia-Lara J. McVicker G. Williams A. Boots M. Foster S. J. Renshaw S. A. (2012). A privileged intraphagocyte niche is responsible for disseminated infection of Staphylococcus aureus in a zebrafish model. Cell. Microbiol. 14, 1600–1619 22694745 10.1111/j.1462-5822.2012.01826.x PMC3470706

[b87-0070785] Prouty M. G. Correa N. E. Barker L. P. Jagadeeswaran P. Klose K. E. (2003). Zebrafish-Mycobacterium marinum model for mycobacterial pathogenesis. FEMS Microbiol. Lett. 225, 177–182 12951238 10.1016/S0378-1097(03)00446-4

[b88-0070785] Ramakrishnan L. (2013). Looking within the zebrafish to understand the tuberculous granuloma. Adv. Exp. Med. Biol. 783, 251–266 23468113 10.1007/978-1-4614-6111-1_13

[b89-0070785] Renshaw S. A. Trede N. S. (2012). A model 450 million years in the making: zebrafish and vertebrate immunity. Dis. Model. Mech. 5, 38–47 22228790 10.1242/dmm.007138 PMC3255542

[b90-0070785] Rigby K. M. DeLeo F. R. (2012). Neutrophils in innate host defense against Staphylococcus aureus infections. Semin. Immunopathol. 34, 237–259 22080185 10.1007/s00281-011-0295-3 PMC3271231

[b91-0070785] Roca F. J. Ramakrishnan L. (2013). TNF dually mediates resistance and susceptibility to mycobacteria via mitochondrial reactive oxygen species. Cell 153, 521–534 23582643 10.1016/j.cell.2013.03.022 PMC3790588

[b92-0070785] Saiman L. Siegel J. (2004). Infection control in cystic fibrosis. Clin. Microbiol. Rev. 17, 57–71 14726455 10.1128/CMR.17.1.57-71.2004 PMC321464

[b93-0070785] Salcedo S. P. Noursadeghi M. Cohen J. Holden D. W. (2001). Intracellular replication of Salmonella typhimurium strains in specific subsets of splenic macrophages in vivo. Cell. Microbiol. 3, 587–597 11553011 10.1046/j.1462-5822.2001.00137.x

[b94-0070785] Sarris M. Masson J. B. Maurin D. Van der Aa L. M. Boudinot P. Lortat-Jacob H. Herbomel P. (2012). Inflammatory chemokines direct and restrict leukocyte migration within live tissues as glycan-bound gradients. Curr. Biol. 22, 2375–2382 23219724 10.1016/j.cub.2012.11.018

[b95-0070785] Schmidtchen A. Frick I. M. Andersson E. Tapper H. Björck L. (2002). Proteinases of common pathogenic bacteria degrade and inactivate the antibacterial peptide LL-37. Mol. Microbiol. 46, 157–168 12366839 10.1046/j.1365-2958.2002.03146.x

[b96-0070785] Shelef M. A. Tauzin S. Huttenlocher A. (2013). Neutrophil migration: moving from zebrafish models to human autoimmunity. Immunol. Rev. 256, 269–281 24117827 10.1111/imr.12124 PMC4117680

[b97-0070785] Sieger D. Peri F. (2013). Animal models for studying microglia: the first, the popular, and the new. Glia 61, 3–9 22987329 10.1002/glia.22385

[b98-0070785] Sieger D. Moritz C. Ziegenhals T. Prykhozhij S. Peri F. (2012). Long-range Ca2+ waves transmit brain-damage signals to microglia. Dev. Cell 22, 1138–1148 22632801 10.1016/j.devcel.2012.04.012

[b99-0070785] Stachura D. L. Traver D. (2011). Cellular dissection of zebrafish hematopoiesis. Methods Cell Biol. 101, 75–110 21550440 10.1016/B978-0-12-387036-0.00004-9

[b100-0070785] Stockhammer O. W. Zakrzewska A. Hegedûs Z. Spaink H. P. Meijer A. H. (2009). Transcriptome profiling and functional analyses of the zebrafish embryonic innate immune response to Salmonella infection. J. Immunol. 182, 5641–5653 19380811 10.4049/jimmunol.0900082

[b101-0070785] Stockhammer O. W. Rauwerda H. Wittink F. R. Breit T. M. Meijer A. H. Spaink H. P. (2010). Transcriptome analysis of Traf6 function in the innate immune response of zebrafish embryos. Mol. Immunol. 48, 179–190 20851470 10.1016/j.molimm.2010.08.011

[b102-0070785] Su F. Juarez M. A. Cooke C. L. Lapointe L. Shavit J. A. Yamaoka J. S. Lyons S. E. (2007). Differential regulation of primitive myelopoiesis in the zebrafish by Spi-1/Pu.1 and C/ebp1. Zebrafish 4, 187–199 18041923 10.1089/zeb.2007.0505

[b103-0070785] Svahn A. J. Graeber M. B. Ellett F. Lieschke G. J. Rinkwitz S. Bennett M. R. Becker T. S. (2013). Development of ramified microglia from early macrophages in the zebrafish optic tectum. Dev. Neurobiol. 73, 60–71 22648905 10.1002/dneu.22039

[b104-0070785] Swaim L. E. Connolly L. E. Volkman H. E. Humbert O. Born D. E. Ramakrishnan L. (2006). Mycobacterium marinum infection of adult zebrafish causes caseating granulomatous tuberculosis and is moderated by adaptive immunity. Infect. Immun. 74, 6108–6117 17057088 10.1128/IAI.00887-06 PMC1695491

[b105-0070785] Takahashi K. Naito M. Takeya M. (1996). Development and heterogeneity of macrophages and their related cells through their differentiation pathways. Pathol. Int. 46, 473–485 8870002 10.1111/j.1440-1827.1996.tb03641.x

[b106-0070785] Tobin D. M. Vary J. C. Jr Ray J. P. Walsh G. S. Dunstan S. J. Bang N. D. Hagge D. A. Khadge S. King M. C. Hawn T. R. (2010). The lta4h locus modulates susceptibility to mycobacterial infection in zebrafish and humans. Cell 140, 717–730 20211140 10.1016/j.cell.2010.02.013 PMC2907082

[b107-0070785] Tobin D. M. Roca F. J. Oh S. F. McFarland R. Vickery T. W. Ray J. P. Ko D. C. Zou Y. Bang N. D. Chau T. T. (2012). Host genotype-specific therapies can optimize the inflammatory response to mycobacterial infections. Cell 148, 434–446 22304914 10.1016/j.cell.2011.12.023 PMC3433720

[b108-0070785] Tsenova L. Bergtold A. Freedman V. H. Young R. A. Kaplan G. (1999). Tumor necrosis factor alpha is a determinant of pathogenesis and disease progression in mycobacterial infection in the central nervous system. Proc. Natl. Acad. Sci. USA 96, 5657–5662 10318940 10.1073/pnas.96.10.5657 PMC21916

[b109-0070785] van der Sar A. M. Musters R. J. van Eeden F. J. Appelmelk B. J. Vandenbroucke-Grauls C. M. Bitter W. (2003). Zebrafish embryos as a model host for the real time analysis of Salmonella typhimurium infections. Cell. Microbiol. 5, 601–611 12925130 10.1046/j.1462-5822.2003.00303.x

[b110-0070785] van der Vaart M. Spaink H. P. Meijer A. H. (2012). Pathogen recognition and activation of the innate immune response in zebrafish. Adv. Hematol. 2012, 159807 22811714 10.1155/2012/159807 PMC3395205

[b111-0070785] van der Vaart M. van Soest J. J. Spaink H. P. Meijer A. H. (2013). Functional analysis of a zebrafish myd88 mutant identifies key transcriptional components of the innate immune system. Dis. Model. Mech. 6, 841–854 23471913 10.1242/dmm.010843 PMC3634667

[b112-0070785] Vandal O. H. Pierini L. M. Schnappinger D. Nathan C. F. Ehrt S. (2008). A membrane protein preserves intrabacterial pH in intraphagosomal Mycobacterium tuberculosis. Nat. Med. 14, 849–854 18641659 10.1038/nmXXXX PMC2538620

[b113-0070785] Vergunst A. C. Meijer A. H. Renshaw S. A. O’Callaghan D. (2010). Burkholderia cenocepacia creates an intramacrophage replication niche in zebrafish embryos, followed by bacterial dissemination and establishment of systemic infection. Infect. Immun. 78, 1495–1508 20086083 10.1128/IAI.00743-09 PMC2849400

[b114-0070785] Volkman H. E. Clay H. Beery D. Chang J. C. Sherman D. R. Ramakrishnan L. (2004). Tuberculous granuloma formation is enhanced by a mycobacterium virulence determinant. PLoS Biol. 2, e367 15510227 10.1371/journal.pbio.0020367 PMC524251

[b115-0070785] Volkman H. E. Pozos T. C. Zheng J. Davis J. M. Rawls J. F. Ramakrishnan L. (2010). Tuberculous granuloma induction via interaction of a bacterial secreted protein with host epithelium. Science 327, 466–469 20007864 10.1126/science.1179663 PMC3125975

[b116-0070785] Wallis R. S. (2008). Tumour necrosis factor antagonists: structure, function, and tuberculosis risks. Lancet Infect. Dis. 8, 601–611 18922482 10.1016/S1473-3099(08)70227-5

[b117-0070785] Wang Y. Curry H. M. Zwilling B. S. Lafuse W. P. (2005). Mycobacteria inhibition of IFN-gamma induced HLA-DR gene expression by up-regulating histone deacetylation at the promoter region in human THP-1 monocytic cells. J. Immunol. 174, 5687–5694 15843570 10.4049/jimmunol.174.9.5687

[b118-0070785] Wittamer V. Bertrand J. Y. Gutschow P. W. Traver D. (2011). Characterization of the mononuclear phagocyte system in zebrafish. Blood 117, 7126–7135 21406720 10.1182/blood-2010-11-321448

[b119-0070785] Xu L. L. Warren M. K. Rose W. L. Gong W. Wang J. M. (1996). Human recombinant monocyte chemotactic protein and other C-C chemokines bind and induce directional migration of dendritic cells in vitro. J. Leukoc. Biol. 60, 365–371 8830793 10.1002/jlb.60.3.365

[b120-0070785] Yang C. T. Cambier C. J. Davis J. M. Hall C. J. Crosier P. S. Ramakrishnan L. (2012). Neutrophils exert protection in the early tuberculous granuloma by oxidative killing of mycobacteria phagocytosed from infected macrophages. Cell Host Microbe 12, 301–312 22980327 10.1016/j.chom.2012.07.009 PMC3638950

[b121-0070785] Yoo S. K. Starnes T. W. Deng Q. Huttenlocher A. (2011). Lyn is a redox sensor that mediates leukocyte wound attraction in vivo. Nature 480, 109–112 22101434 10.1038/nature10632 PMC3228893

[b122-0070785] Zakrzewska A. Cui C. Stockhammer O. W. Benard E. L. Spaink H. P. Meijer A. H. (2010). Macrophage-specific gene functions in Spi1-directed innate immunity. Blood 116, e1–e11 20424185 10.1182/blood-2010-01-262873

