# Supplementary material for: Macrophage-pathogen interactions in infectious diseases: new therapeutic insights from the zebrafish host model
Source: Dis Model Mech. 2014 Jul;7(7):785–97. doi: 10.1242/dmm.015594 (PMC4073269; doi:10.1242/dmm.015594)
Supplement: Supplementary Material [file supp_7_7_785__index.html]

Supplementary Material 

# Macrophage-pathogen interactions in infectious diseases: new therapeutic insights from the zebrafish host model

## DMM015594 Supplementary Material

**Files in this Data Supplement:**

- **Supplementary Material**
