# Supplementary material for: Macrophage-pathogen interactions in infectious diseases: new therapeutic insights from the zebrafish host model
Source: Dis Model Mech. 2014 Jul;7(7):785–97. doi: 10.1242/dmm.015594 (PMC4073269; doi:10.1242/dmm.015594)
Supplement: Supplementary Material [file supp_7.7.785_DMM015594.pdf]

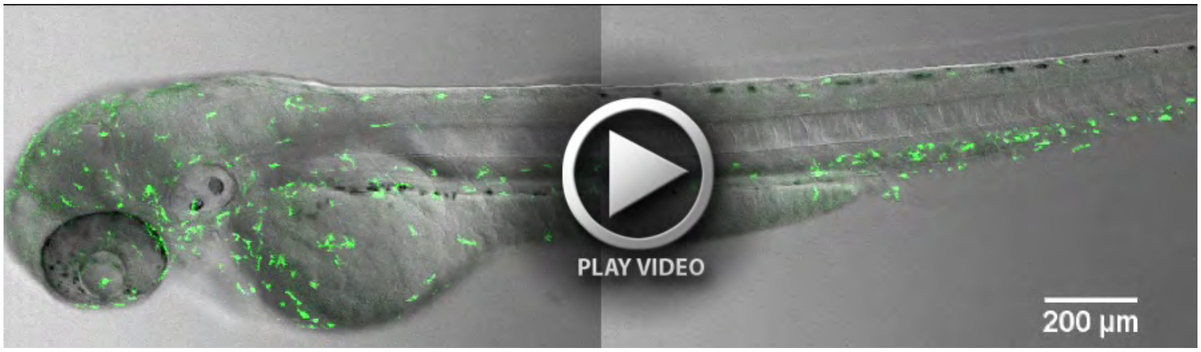

Supplementary Movie 1. Random patrolling of macrophages in a *Tg(mpeg1:Gal4-VP16/UAS-E1b:Kaede)* zebrafish larva. See Fig. 2A for details. Scale bar reported in the first movie stack (200 µm).

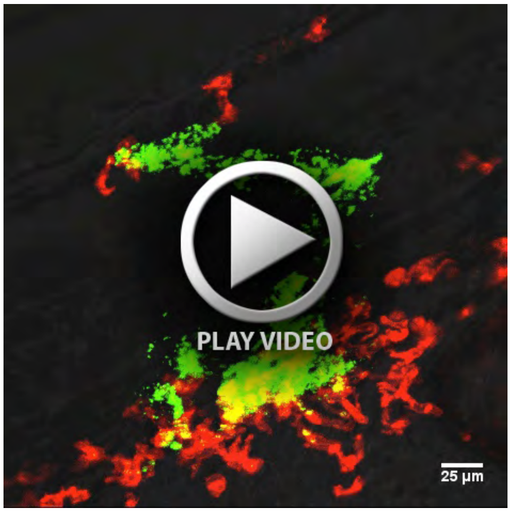

Supplementary Movie 2. Phagocytosis of GFP-labeled *M. marinum in Tg(mpeg1:mCherry-F)* macrophage reporter line. See Fig. 2B for details. Note that bacteria are also clustered in non-labeled cells, most likely neutrophils. Scale bar reported in the first movie stack (25 µm).

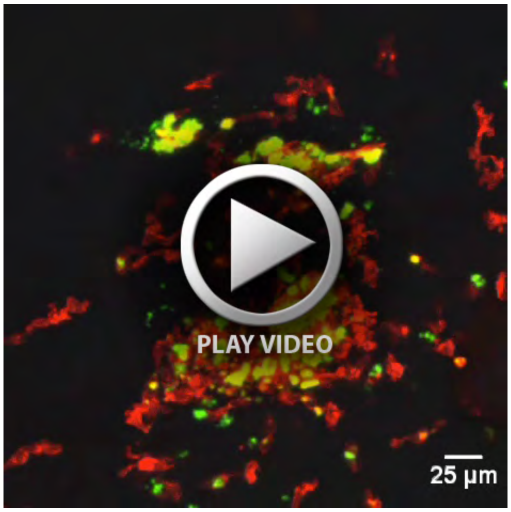

Supplementary Movie 3. Dissemination of GFP-labeled *M. marinum* by macrophages in *Tg(mpeg1:mCherry-F)* reporter line. See Fig. 2C for details. As in supplementary material Movie 1, note that bacteria are also clustered in non-labeled cells, most likely neutrophils. Scale bar reported in the first movie stack (25 µm).

Supplementary material Table S1. Zebrafish fluorescent reporter lines for macrophages.

Marker	Transgenic line	Specificity	Promoter sequence	Reference
apoeb<br>apolipoprotein Eb	*Tg(apoeb:lynEGFP)*	Microglial cells	Obtained from a BAC clone containing the *apoeb* locus, by BAC recombineering at the translation start	Peri and Nüsslein-Volhard, 2008
coro1a<br>coronin, actin binding protein, 1A	*Tg(coro1a:EGFP)*	Macrophages, neutrophils and thymocytes[(1)]	-7.03-kb of *coro1a* upstream the translation start	Li et al., 2012
csf1ra/fms<br>colony stimulating factor 1 receptor, a	*TgBAC(csf1ra:Gal4-VP16)/ Tg(UAS-E1b:Eco.NfsB-mCherry)*	Macrophages (highly motile), and xantophore cells (immobile)	Obtained from the BAC clone HUKGB735K06247Q containing the *csf1ra* locus, by BAC recombineering at the translation start	Gray et al., 2011
fli1a<br>friend leukemia integration 1a	*Tg(fli1a:EGFP)*	Primitive macrophages (dull), endothelial cells (bright) and subsets of erythrocytes (dull)	5'UTR of *fli* was obtained from a PAC library	Lawson and Weinstein, 2002
lyz/lysC<br>lysozyme	*Tg(lyz:EGFP)*	Neutrophils[(2)]	-11 kb of *lyz* upstream the translation start	Hall et al., 2007
	Tg(lyz:DsRed2)	Neutrophils[(2)]	-6.35 kb of *lyz* upstream the translation start	Hall et al., 2007
	Tg(-4.1lyz:EGFP)	Neutrophils[(2)]	-4.1 kb of *lyz* upstream the translation start	Zhang et al., 2008
	Tg(-2.4lyz:EGFP)	Neutrophils and primitive macrophages[(3)]	-2.4 kb of *lyz* upstream the translation start	Kitaguchi et al., 2009
	Tg(lyz:Gal4-VP16)	Neutrophils[(2)]	-11 kb of *lyz* construct as in *Hall, 2007*	Elks et al., 2011
mhc2dab<br>major histocompatibility complex class II DAB gene	*Tg(mhc2dab:EGFP)*<br>*Tg(mhc2dab:mCherry)*	Antigen presenting cells (APCs)[(4)]	-3.8 kb of *mhc2dab* upstream the transcription start	Wittamer et al., 2011
mpeg1<br>macrophage expressed 1	*Tg(mpeg1:EGFP)*<br>*Tg(mpeg1:mCherry)*<br>*Tg(mpeg1:Gal4-VP16)*	Macrophages[(5)]	-1.86 kb of *mpeg1* upstream the translation start	Ellett et al., 2011
	Tg(mpeg1:mCherry-F)	Macrophages	-1.86 kb of *mpeg1* upstream the translation start	Bernut et al., 2014
	Tg(mpeg1:Dendra2)	Macrophages[(5)]	As in *Ellet et al., 2011*	Harvie et al., 2013
	Tg(mpeg1:YFP)	Macrophages[(5)]	As in *Ellet et al., 2011*	Roca and Ramakrishnan, 2013
mpx/mpo<br>myeloid-specific peroxidase	*TgBAC(mpx:EGFP)*	Neutrophils[(6)]	Obtained from the BAC clone zC91B8 containing the *mpx locus,* by BAC recombineering at the translation start	Renshaw et al., 2006
	Tg(mpx:GFP)	Neutrophils (bright) and a subset of macrophages (dull)	-8 kb of *mpx* upstream the translation start	Mathias et al., 2006
	Tg(-8mpx:mCherry)<br>*Tg(-8mpx:DsRed-F)*<br>*Tg(-8 mpx:EGFP-F)*	Neutrophils (bright) and a subset of macrophages (dull)	As in *Mathias, 2006*	Yoo et al., 2010
	Tg(-8mpx:Dendra2)	Neutrophils (bright) and a subset of macrophages (dull)	As in *Mathias, 2006*	Yoo and Huttenlocher, 2011
myd88<br>myeloid differentiation primary response gene (88)	*Tg(myd88:EGFP)*<br>*Tg(myd88:DsRed2)*	Subsets of myeloid leukocytes, distal pronephric ducts and cloaca	-3.7 kb of *myd88* upstream the translation start	Hall et al., 2009
ptprc/cd45<br>protein tyrosine phosphatase, receptor type, C	*Tg(ptprc:DsRed)*	Macrophages, granulocytes and T lymphocytes	-7.6 kb of *ptprc* upstream the transcription start	Bertrand et al., 2008
spi1b/pu.1<br>spleen focus forming virus (SFFV) proviral integration oncogene spi1b	*Tg(-5.3spi1b:EGFP)*	Early myeloid cells[(7)]	-5.3 kb of *spi1b* upstream the translation start	Ward et al., 2003
	Tg(-9.0spi1b:EGFP)	Early myeloid cells (bright) and muscles (dull)[(7)]	-9 kb of *spi1b* upstream the translation start	Hsu et al., 2004
	Tg(-4spi1b:Gal4/UAS:EGFP)	Early myeloid cells[(7)]	-4 kb of *spi1b* upstream the translation start	Peri and Nüsslein-Volhard, 2008
	Tg(-4spi1:lynEGFP)	Early myeloid cells[(7)]	As in *Peri and Nüsslein-Volhard 2008*	Zakrzewska et al., 2010

Notes:

(1) Expression in macrophages, neutrophils and thymocytes was shown for embryonic and young larval stages. Expression in myelomonocyte progenitors in head-kidneys of adults was also documented.

(2) Discrepancies about expression of the construct in primitive macrophages reported.

(3) Observations limited to embryonic and early larval stage. Labeling of primitive macrophages was determined based on the presence of fluorescent cells over the yolk at 26 hpf. Expression in macrophages from the second wave of primitive haemopoiesis and definitive haemopoiesis not demonstrated.

(4) APCs are here indicated as macrophages, dendritic cells, B lymphocytes and eosinophils. Fluorescence visible from 5dpf and abundantly labeling APCs only from 12dpf; expression in keratinocytes reported in adults and juveniles.

(5) Loss of expression after 6 dpf and in the adults reported; labeling of Langerhans dendridic cells in larvae suggested.

(6) Existence of a set of GFP^{low} macrophage-like cells distinguishable by confocal microscopy reported by some laboratories.

(7) Expression documented from 12 to 30 hpf; Expression in early lymphoid cells reported for adult *Tg(-9.0spi1b:EGFP).*

Supplementary table 1. References

Bernut, A., Herrmann, J. L., Kissa, K., Dubremetz, J. F., Gaillard, J. L., Lutfalla, G. and Kremer, L. (2014). Mycobacterium abscessus cording prevents phagocytosis and promotes abscess formation. *Proc Natl Acad Sci U S A* **111**, E943-952.

Bertrand, J. Y., Kim, A. D., Teng, S. and Traver, D. (2008). CD41+ cmyb+ precursors colonize the zebrafish pronephros by a novel migration route to initiate adult hematopoiesis. *Development* **135**, 1853-1862.

Elks, P. M., van Eeden, F. J., Dixon, G., Wang, X., Reyes-Aldasoro, C. C., Ingham, P. W., Whyte, M. K., Walmsley, S. R. and Renshaw, S. A. (2011). Activation of hypoxia-inducible factor-1alpha (Hif-1alpha) delays inflammation resolution by reducing neutrophil apoptosis and reverse migration in a zebrafish inflammation model. *Blood* **118**, 712-722.

Ellett, F., Pase, L., Hayman, J. W., Andrianopoulos, A. and Lieschke, G. J. (2011). mpeg1 promoter transgenes direct macrophage-lineage expression in zebrafish. *Blood* **117**, e49-56.

Gray, C., Loynes, C. A., Whyte, M. K., Crossman, D. C., Renshaw, S. A. and Chico, T. J. (2011). Simultaneous intravital imaging of macrophage and neutrophil behaviour during inflammation using a novel transgenic zebrafish. *Thromb Haemost* **105**, 811-819.

Hall, C., Flores, M. V., Storm, T., Crosier, K. and Crosier, P. (2007). The zebrafish lysozyme C promoter drives myeloid-specific expression in transgenic fish. *BMC Dev Biol* **7**, 42.

Hall, C., Flores, M. V., Chien, A., Davidson, A., Crosier, K. and Crosier, P. (2009). Transgenic zebrafish reporter lines reveal conserved Toll-like receptor signaling potential in embryonic myeloid leukocytes and adult immune cell lineages. *J Leukoc Biol* **85**, 751-765.

Harvie, E. A., Green, J. M., Neely, M. N. and Huttenlocher, A. (2013). Innate immune response to Streptococcus iniae infection in zebrafish larvae. *Infect Immun* **81**, 110-121.

Hsu, K., Traver, D., Kutok, J. L., Hagen, A., Liu, T. X., Paw, B. H., Rhodes, J., Berman, J. N., Zon, L. I., Kanki, J. P. et al. (2004). The pu.1 promoter drives myeloid gene expression in zebrafish. *Blood* **104**, 1291-1297.

Kitaguchi, T., Kawakami, K. and Kawahara, A. (2009). Transcriptional regulation of a myeloid-lineage specific gene lysozyme C during zebrafish myelopoiesis. *Mech Dev* **126**, 314-323.

Lawson, N. D. and Weinstein, B. M. (2002). In vivo imaging of embryonic vascular development using transgenic zebrafish. *Dev Biol* **248**, 307-318.

Li, L., Yan, B., Shi, Y. Q., Zhang, W. Q. and Wen, Z. L. (2012). Live imaging reveals differing roles of macrophages and neutrophils during zebrafish tail fin regeneration. *J Biol Chem* **287**, 25353-25360.

Mathias, J. R., Perrin, B. J., Liu, T. X., Kanki, J., Look, A. T. and Huttenlocher, A. (2006). Resolution of inflammation by retrograde chemotaxis of neutrophils in

transgenic zebrafish. *J Leukoc Biol* **80**, 1281-1288.

Peri, F. and Nusslein-Volhard, C. (2008). Live imaging of neuronal degradation by microglia reveals a role for v0-ATPase a1 in phagosomal fusion in vivo. *Cell* **133**, 916-927.

Renshaw, S. A., Loynes, C. A., Trushell, D. M., Elworthy, S., Ingham, P. W. and Whyte, M. K. (2006). A transgenic zebrafish model of neutrophilic inflammation. *Blood* **108**, 3976-3978.

Roca, F. J. and Ramakrishnan, L. (2013). TNF dually mediates resistance and susceptibility to mycobacteria via mitochondrial reactive oxygen species. *Cell* **153**, 521-534.

Ward, A. C., McPhee, D. O., Condron, M. M., Varma, S., Cody, S. H., Onnebo, S. M., Paw, B. H., Zon, L. I. and Lieschke, G. J. (2003). The zebrafish spi1 promoter drives myeloid-specific expression in stable transgenic fish. *Blood* **102**, 3238-3240.

Wittamer, V., Bertrand, J. Y., Gutschow, P. W. and Traver, D. (2011). Characterization of the mononuclear phagocyte system in zebrafish. *Blood* **117**, 7126-7135.

Yoo, S. K. and Huttenlocher, A. (2011). Spatiotemporal photolabeling of neutrophil trafficking during inflammation in live zebrafish. *J Leukoc Biol* **89**, 661-667.

Yoo, S. K., Deng, Q., Cavnar, P. J., Wu, Y. I., Hahn, K. M. and Huttenlocher, A. (2010). Differential regulation of protrusion and polarity by PI3K during neutrophil motility in live zebrafish. *Dev Cell* **18**, 226-236.

Zakrzewska, A., Cui, C., Stockhammer, O. W., Benard, E. L., Spaink, H. P. and Meijer, A. H. (2010). Macrophage-specific gene functions in Spi1-directed innate immunity. *Blood* **116**, e1-11.

Zhang, Y., Bai, X. T., Zhu, K. Y., Jin, Y., Deng, M., Le, H. Y., Fu, Y. F., Chen, Y., Zhu, J., Look, A. T. et al. (2008). In vivo interstitial migration of primitive macrophages mediated by JNK-matrix metalloproteinase 13 signaling in response to acute injury. *J Immunol* **181**, 2155-2164.

Supplementary material Table S2. Zebrafish reporters of subcellular compartments.

Marker	Reporter construct	Specificity	Notes	Reference
**rab5c** RAB5c, member RAS oncogene family	*Tg(h2afx:EGFP-rab5c)*(1)	Early endosomes	-	Clark et al., 2011
	Tg(UAS:mCherry-rab5c_S36N)(2)	Early endosomes	S36N substitution, dominant negative	Clark et al., 2011
	Tg(UAS:mCherry-rab5c_Q81L)(2)	Early endosomes	Q81L substitution, constitutively active	Clark et al., 2011
rab7a RAB7, member RAS oncogene family a	*Tg(h2afx:EGFP-rab7)*(1)	Late endosomes	-	Clark et al., 2011
	Tg(UAS:mCherry-rab7_T22N)(2)	Late endosomes	T22N substitution, dominant negative	Clark et al., 2011
	Tg(UAS:mCherry-rab7_Q67L)(2)	Late endosomes	Q67L substitution, constitutively active	Clark et al., 2011
rab7b RAB7, member RAS oncogene family b	*Plasmid(UAS:GFP-rab7b_T22N)*(2)	Late endosomes	T22N substitution, dominant negative; the reporter consists of an UAS-expression plasmid; transgenic line not available	Ellis et al., 2013
rab11a RAB11a, member RAS oncogene family	*Tg(h2afx:EGFP-rab11a)*(1)	Recycling endosomes	-	Clark et al., 2011
	Tg(UAS:mCherry-rab11a_S25N)(2)	Recycling endosomes	S25N substitution, dominant negative	Clark et al., 2011
	Tg(UAS:mCherry-rab11a_Q70L)(2)	Recycling endosomes	Q70L substitution, constitutively active	Clark et al., 2011
lamp1 lysosomal-associated membrane protein 1	*Tg(hsp70l:lamp1-RFP)*(3)	Lysosomes	-	Ellis et al., 2013
lamp2 lysosomal membrane glycoprotein 2	*Tg(hsp70l:lamp2-EGFP)*(3)	Lysosomes	-	Ellis et al., 2013
cd63/lamp3 Cd63 antigen	*Plasmid(CMV/SP6:GFP-cd63)*(4)	Lysosomes	The reporter consists of an expression plasmid for CMV-expression or mRNA preparation; transgenic line not available	Peri and Nüsslein-Volhard, 2008
map1lc3b microtubule-associated protein 1 light chain 3 beta	*Tg(CMV:EGFP-map1lc3b)*(4)	Autophagosomes	-	He et al., 2009
	Tg(hsp70l:RFP-Rno.Map1lc3b)(3)	Autophagosomes	Contains the Map1lc3b sequence from the rat (*Rattus norvegicus*)	Ellis et al., 2013
gabarapa GABA(A) receptor-associated protein a	*Tg(CMV:EGFP-gabarapa)*(4)	Autophagosomes	-	He et al., 2009
rab32a RAB32a, member RAS oncogene family	*Tg(4xUAS:EGFP-rab32a,myl7:EGFP)*(2)	Vesicular trafficking	-	Ellis et al., 2013
	Plasmid(UAS:GFP-rab32a_T27N)(2)	Vesicular trafficking	T27N substitution, dominant negative; the reporter consists of an UAS-expression plasmid; transgenic line not available	Ellis et al., 2013
rab38b	*Tg(UAS:GFP-rab38b-T23N, cmlc2:GFP)*(2)	Vesicular trafficking	T23N substitution, dominant negative	Ellis et al., 2013

Notes:
(1) *h2afx*: H2A histone family, member X constitutive promoter
(2) *UAS*: Upstream Activating Sequence for Gal4-dependent expression
(3) *hsp70l*: heat shock cognate 70-kd protein, like inducible promoter
(4) *CMV*: Cytomegalovirus constitutive promoter

Supplementary table 2. References

Clark, B. S., Winter, M., Cohen, A. R. and Link, B. A. (2011). Generation of Rab-based transgenic lines for in vivo studies of endosome biology in zebrafish. *Dev Dyn* **240**, 2452-2465.

Ellis, K., Bagwell, J. and Bagnat, M. (2013). Notochord vacuoles are lysosome-related organelles that function in axis and spine morphogenesis. *J Cell Biol* **200**, 667-679.

He, C., Bartholomew, C. R., Zhou, W. and Klionsky, D. J. (2009). Assaying autophagic activity in transgenic GFP-Lc3 and GFP-Gabarap zebrafish embryos. *Autophagy* **5**, 520-526.

Peri, F. and Nusslein-Volhard, C. (2008). Live imaging of neuronal degradation by microglia reveals a role for v0-ATPase a1 in phagosomal fusion in vivo. *Cell* **133**, 916-927.
